# TRIM4 enhances small-molecule-induced neddylated-degradation of CORO1A for triple negative breast cancer therapy

**DOI:** 10.7150/thno.97662

**Published:** 2024-10-21

**Authors:** Wen-Jie Gu, Xiao-Xia Liu, Yi-Wen Shen, Yi-Ting Gong, Yi-Li Chen, Jiayi Lin, Dong Lu, Li-Jun Zhang, Hong-Zhuan Chen, Yi Jin, Zha-Jun Zhan, Wei-Dong Zhang, Jin-Mei Jin, Xin Luan

**Affiliations:** 1 Shanghai Frontiers Science Center for Chinese Medicine Chemical Biology; Institute of Interdisciplinary Integrative Medicine Research and Shuguang Hospital; Shanghai University of Traditional Chinese Medicine, Shanghai, 201203, China.; 2 College of Pharmaceutical Science, Zhejiang University of Technology, Hangzhou, 310014, China.; 3 State Key Laboratory for Quality Ensurance and Sustainable Use of Dao-di Herbs, Institute of Medicinal Plant Development, Chinese Academy of Medical Science and Peking Union Medical College, Beijing, 100700, China.; 4 School of Pharmacy, Second Military Medical University, Shanghai, 200433, China.; 5 Key Laboratory of Medicinal Chemistry for Natural Resource, Ministry of Education; Yunnan Provincial Center for Research & Development of Natural Products; School of Chemical Science and Technology, Yunnan University, Kunming 650091, China.

**Keywords:** Triple-negative breast cancer, CORO1A, Aurovertin B, Molecular glues, Neddylation

## Abstract

**Background:** As a critical member of the Coronin family, Coronin 1A (CORO1A) plays a crucial role in the progression of triple-negative breast cancer (TNBC). However, CORO1A is typically considered “undruggable” due to its smooth surface and complex protein-protein interactions (PPIs). Molecular glues have emerged as one of the most effective strategies to rapidly degrade such “undruggable” targets. Neddylation, an emerging approach, has shown promise in targeting pathogenic proteins for degradation through the NEDD8 pathway, making the degradation of CORO1A an attractive pharmacological strategy.

**Methods:** A phenotypic drug screening strategy coupled with multi-omics approaches was utilized to rapidly identify a molecular glue degrader for CORO1A and to uncover the associated mechanisms. The Omics and Text-based Target Enrichment and Ranking (OTTER) tools, co-immunoprecipitation (Co-IP) assay, mass spectrometry, and the separation of phases-based protein interaction reporter (SPPIER) method were employed to explore the interaction between Aurovertin B (AB) and CORO1A via TRIM4. The pharmacological effects of AB were assessed using TNBC patient-derived organoids (PDOs) and 3D bioprinting models.

**Results:** We identified AB as a previously undisclosed molecular glue that significantly promotes the neddylation and proteasomal degradation of CORO1A via TRIM4, an atypical E3 ligase. Notably, the degradation of CORO1A markedly inhibited various cellular processes and exerted robust antitumor effects in TNBC PDOs and 3D bioprinting models.

**Conclusions:** Our findings underscore the critical role of CORO1A in TNBC and lay a crucial foundation for the development of innovative drugs based on molecular glue technology.

## Introduction

Breast cancer (BC) is the leading cause of cancer-related deaths in women, ranking first in incidence and second in mortality worldwide 1. Approximately 15-25% of all BC cases lack the biomarkers human epidermal growth factor receptor 2 (HER2), estrogen receptor (ER), and progesterone receptor (PR), classifying them as triple-negative breast cancer (TNBC). TNBC is characterized by high recurrence and metastasis rates, and poor clinical outcomes 2,3. Current treatment options for TNBC primarily include chemotherapy, radiotherapy (RT), and immunotherapy 3. However, these treatments often cause undesirable side effects, damage to healthy tissues, and are hindered by the lack of clear and universal molecular targets, making TNBC treatment particularly challenging 3,4. Notably, 40% of TNBC tumors tend to relapse, developing drug resistance and metastatic features, resulting in 80%-90% of TNBC patients dying mainly from metastasis. This underscores the urgent need to develop new therapeutic targets or approaches for TNBC 3.

The Coronin family comprises seven evolutionarily conserved proteins that regulate a wide array of biological activities 5. Recent studies on oncogenic functions have highlighted the critical role of Coronins in tumor progression and prognosis 6-8. Coronin 1A (CORO1A), a 57 kDa protein, is a key member of this family and is closely associated with tumor migration 6,9. Research shows that CORO1A is persistently activated in various invasive tumors, such as TNBC, hepatocellular carcinomas, and melanomas, compared to benign tumors or normal tissues 6,10. Furthermore, the aberrant overexpression of CORO1A is linked to increased tumor malignancy, and preclinical evidence strongly supports CORO1A as a biomarker for cancer metastasis 6,11. Although an antibody targeting CORO1A has been developed, its application has been limited to B-lineage malignancies, and its pharmacodynamics in solid tumors has not been explored 12. Challenges in developing siRNAs to target CORO1A include limited bioavailability and poor cell permeability 13. Additionally, CORO1A's numerous and complex protein-protein interactions (PPIs) contribute to cell motility and actin dynamics, making it difficult for small molecule inhibitors to completely inhibit its functional sites 14. The presence of multiple phosphorylation sites further complicates the development of effective CORO1A inhibitors 15.

The targeted protein degradation (TPD) strategy offers a novel therapeutic approach through proteasomal or lysosomal pathways 13,16. Molecular glues, which have smaller molecular weights, improved cellular permeability, and increased oral bioavailability, have emerged as a promising approach to degrade specific disease-associated proteins that lack druggable pockets by exploiting the cell's destruction machinery 17-19. Several molecular glues have entered clinical trials and demonstrated good efficacy. Mechanistically, the molecular glue mainly acts as a linker to connect specific E3 ubiquitin ligase and substrate protein, inducing the ubiquitination and degradation of target proteins 19. A prime example is thalidomide, which targets the E3 ubiquitin ligase CRBN, leading to the polyubiquitination and subsequent degradation of IKZF3 18. Like ubiquitination, neddylation is a posttranslational modification that conjugates the ubiquitin-like protein NEDD8 to target proteins, promoting the activation of RING E3 complexes 20. In 2020, Kheewoong Baek *et al.* found that NEDD8 nucleated the multivalent ubiquitin ligation assembly, initiating the ubiquitination degradation process 20.

In this study, we combined a phenotypic drug screening strategy with multi-omics approaches to rapidly identify a molecular glue degrader for CORO1A and elucidate the underlying mechanisms. We found that Aurovertin B (AB) impaired TNBC cell viability in a neddylation and proteasome-dependent manner. Proteomic profiling and transcriptome sequencing further demonstrated the interaction between AB and the E3 ubiquitin-conjugating enzyme TRIM4. Notably, the formation of the CORO1A-AB-TRIM4 ternary complex led to significant degradation of CORO1A, resulting in pronounced inhibitory effects both *in vitro* and *in vivo*, especially in TNBC PDO models and 3D bioprinting models. Collectively, this study highlights a molecular glue-based strategy for targeting CORO1A in TNBC therapy.

## Results

### CORO1A overexpression displays poor clinical prognosis in TNBC patients

**Figure 1:**
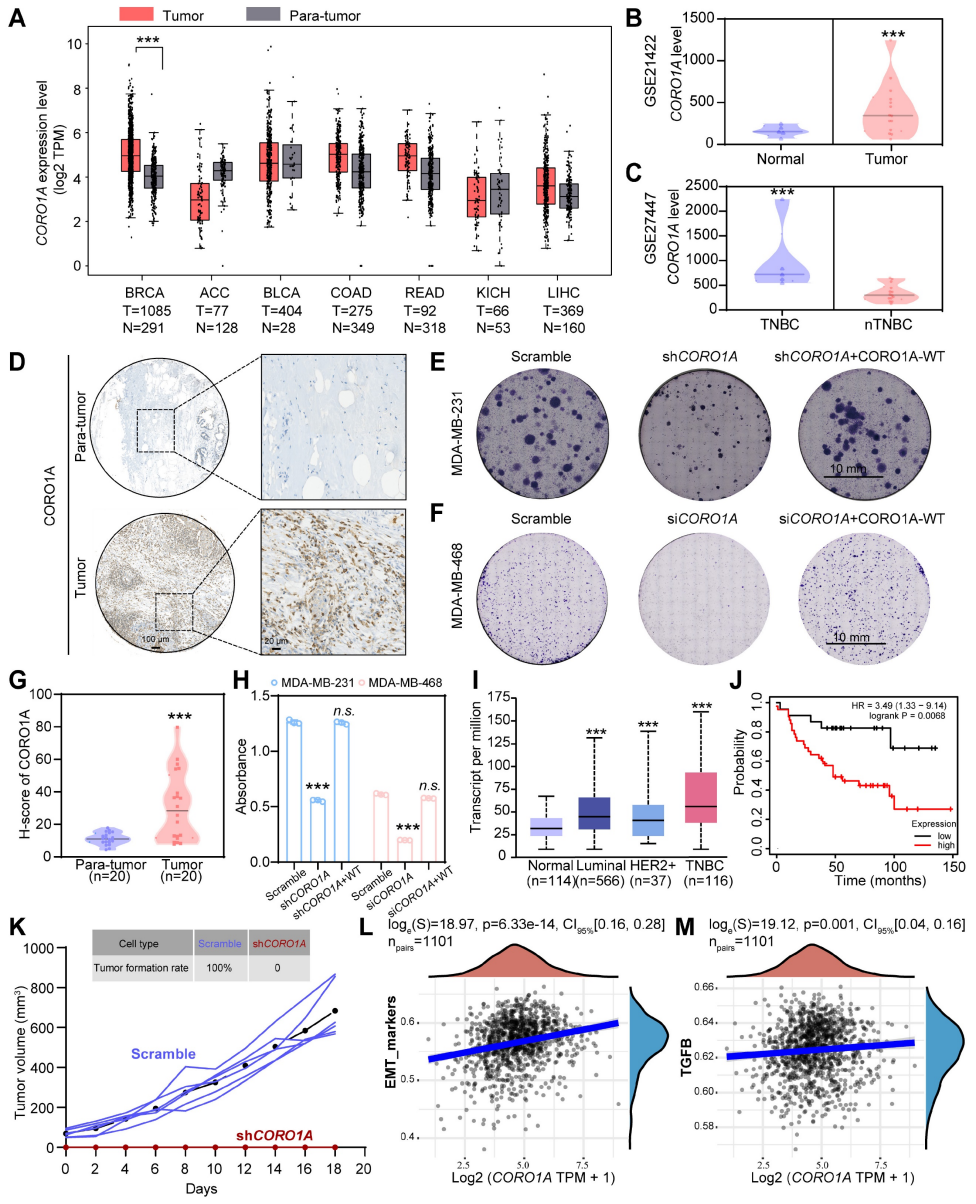
CORO1A overexpression correlated with poor clinical outcomes in TNBC patients. **(A)** Pan-cancer analysis of *CORO1A* using the GEPIA database. *CORO1A* expression was significantly increased in BC patients using the GSE21422 database **(B)**. **(C)*** CORO1A* expression was significantly increased in patients with TNBC (GSE27447 database). **(D)** CORO1A overexpression was observed in TNBC patients using tissue chip staining. The H-score of CORO1A was showed in **(G)**. Representative images of colony formation were shown in MDA-MB-231 cells with sh*CORO1A* **(E)** and in MDA-MB-468 cells with si*CORO1A*** (F)**. The analysis was showed in **(H)** compared to the Scramble group. **(I)** The expression of *CORO1A* in different subtypes of BC was higher than that in normal tissues, especially in TNBC. **(J)** A high level of* CORO1A* was correlated with poor prognosis in TNBC patients.** (K)** Tumorigenicity experiments showed that the MDA-MB-231 cells with sh*CORO1A* failed to promote tumor growth. The EMT **(L)** and TGFβ pathway** (M)** scores correlated highly with *CORO1A* across BC tumors. Data were presented as mean ± SD, ***P ˂ 0.001.

Elevated levels of Coronins have been reported in various solid and hematological tumors compared to adjacent normal mucosa tissues, which is closely associated with malignant pathological features and poor clinical outcomes in different stages, indicating the potential of Coronins as valuable prognostic indicator 9,15. To assess the clinical implications of CORO1A in TNBC, the bioinformatic analysis was employed using the public GEPIA and GEO databases. As we can see, *CORO1A* expression in breast invasive carcinoma (BRCA) tumor samples was higher than that in normal tissues (Figure 1A). Consistently, the analysis of the GSE21422 database revealed that the *CORO1A* level in tumor samples was higher than that in normal tissues (Figure 1B, Figure S1A). Moreover, we found that *CORO1A* expression in TNBC patients was significantly higher than that of non-TNBC (nTNBC) patients (Figure 1C, Figure S2A). Furthermore, the immunohistochemical (IHC) staining of 20 pairs of the biopsy specimens from clinical TNBC patients through the tissue chip identified a higher content of CORO1A compared with adjacent tissues (Figure 1D, G). To further elucidate the crucial role of CORO1A in TNBC carcinogenesis, we constructed a lentiviral shRNA and the si*CORO1A* to knockdown *CORO1A* in TNBC cells. As showed in Figure S3, the sh*CORO1A*-3 and siRNA-2 had good effects and were used for next experiments. When the CORO1A protein expression was silenced compared with scramble (Figure S3A-B), the TNBC cells exhibited an attenuated pro-proliferation ability by forming fewer colonies (Figure 1E- F, H). However, the proliferation and cell viability of TNBC cells were restored after CORO1A supplementation (Figure 1E-F, H, S4).

To examine whether tumors with high or low CORO1A were clinically distinct, we further determined the *CORO1A* expression in different subtypes of BC using TCGA database. Significantly, the increased expression of *CORO1A* was inversely correlated with the prognosis of TNBC patients (Figure 1I-J). To further verify the effects of cell proliferation ability in non-TNBC cell lines after CORO1A knockdown, we used the siRNA-2 to knockdown *CORO1A* in MCF7 cells. The results showed that the cell proliferation ability was barely affected (Figure S5A). In addition, the supplementation of CORO1A in the MCF7 si*CORO1A* group also had no significant effect on cell viability (Figure S5B). *In vivo,* tumorigenicity experiments also showed that the MDA-MB-231 cells with sh*CORO1A* failed to promote tumor growth (Figure 1K). The correlation scatter plots demonstrated that *CORO1A* expression was positively correlated with the expression of the epithelial-mesenchymal transition (EMT)-related TGF-β signaling pathway (Figure 1L-M). We then knocked down *CORO1A* using siRNA-2 and performed the transcriptome sequencing assay again. KEGG enrichment analysis and GSEA analysis showed the significantly decreased TGF-β signaling pathways (Figure S6A-C). We also found that the expression of key genes in the TGF-β/Smads and EMT pathways was significantly reduced, indicating that *CORO1A* plays roles in EMT and TGF-β signaling pathway (Figure S6D). Collectively, these data suggested that the CORO1A overexpression had unfavorable impact on patient outcomes and may serve as prognostic biomarker and potential therapeutic target in TNBC.

### AB was repurposed as the first small-molecule inhibitor bounding to CORO1A

**Figure 2:**
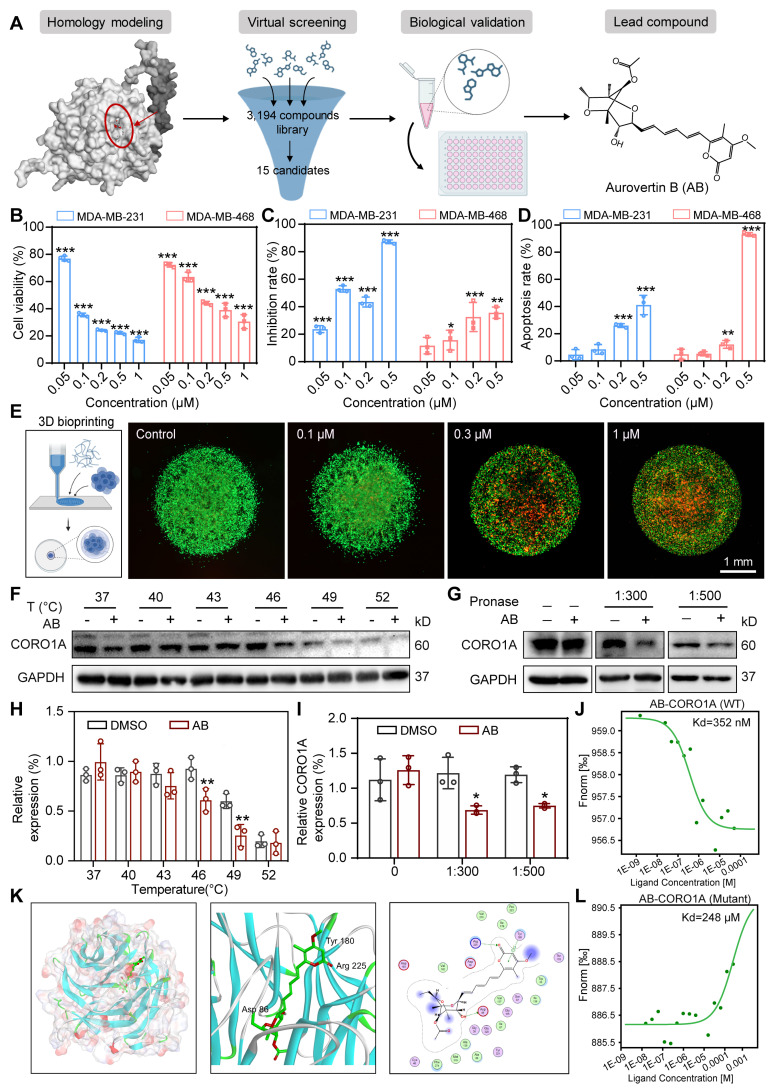
AB exerted anti-TNBC effects by directly binding to CORO1A.** (A)** The pattern diagram of virtual screening and the chemical structure of AB. **(B)** Cell viability of MDA-MB-231 and MDA-MB-468 cells after AB treatment for 24 h compared to group with no drug treatment. **(C)** Transwell assay showed that AB inhibited cell migration compared with no drug treatment. **(D)** A flow cytometry assay showed AB-induced cell apoptosis compared with no drug treatment. **(E)** Representative images of Live/Dead staining of cells with 3D bioprinting. **(F)** CETSA assays verified the destabilization of CORO1A in MDA-MB-231 cells. **(G)** DARTS assays confirmed the destabilization of CORO1A during proteolysis in MDA-MB-231 cells. **(H)** Analysis of **(F)**. **(I)** Analysis of** (G)**. **(J)** MST analysis of the binding of GFP-tagged CORO1A with AB. **(K)** Molecular docking results of AB binding to CORO1A.** (L)** MST analysis of the binding of GFP-tagged mutant CORO1A with AB. Data were presented as mean ± SD, *P ˂ 0.05, **P ˂ 0.01, ***P ˂ 0.001 versus the control group.

As the inhibition of CORO1A resulted in the regression of TNBC, we next used the virtual screening library containing 3194 compounds, followed by cytotoxic assays to identify candidate compounds that specifically target CORO1A (Figure 2A). The top 15 candidates with higher docking scores were selected for further assay (Table S1). Among them, Aurovertin B (AB) showed the best antitumor effect in TNBC cells (Figure 2A, Figure S7, Table S1). As the natural compound isolated from Calcarisporium arbuscula (Figure 2A), AB exhibited an outstanding anti-tumor effect in BC 21, while the specific mechanisms remained unclear. To identify the inhibitory impacts of AB on TNBC cell viability, a panel of TNBC cell lines, other types of tumor cell lines, and one normal mammary epithelial cell line MCF-10A were treated with AB for 24 h. As shown in Figure S8, AB significantly inhibited the growth of TNBC cell lines, especially in MDA-MB-231 and MDA-MB-468 cells in a dose-dependent manner (Figure 2B, Figure S9A), and its cytotoxicity was much more significant than that in MCF-10A, indicating the excellent selectivity of AB. The Nanolive assay was further performed to visualize the cell morphology after AB treatment. We observed that AB rapidly induced the loss of cell morphology and the rupture of contents in MDA-MB-231 and MDA-MB-468 cells (Figure S9B-C). We further performed the wound healing and transwell assays to confirm that the horizontal (Figure S10A-B) and longitudinal migration (Figure 2C, Figure S10C) ability of the TNBC cells were significantly inhibited through AB. Flow cytometry assay also showed that AB induced cell apoptosis (Figure 2D, Figure S10D) and inhibited reactive oxygen species (ROS) (Figure S10E-G, S11) production. Real-time cell analysis (RTCA) assays proved that AB showed potent inhibition on the cell proliferation capacity of MDA-MB-231 (Figure S10H) and MDA-MB-468 (Figure S10I) cells. Recent advances in 3D bioprinting provide a valuable tool to fabricate biomimetic constructs, which can be applied in different stages of drug discovery research. To further investigate the clinical application potential of AB, we used 3D printing technology to construct MDA-MB-231 spheroids (Figure 2E), and subsequently, the LIVE/DEAD assay was performed. 3D bioprinting assay also showed that AB significantly impaired the viability of MDA-MB-231 spheroids (Figure 2E).

Based on the potent anti-tumor efficacy of AB in TNBC cells, we next performed drug affinity response target stability (DARTS) combined with mass spectrometry (DARTS-MS) strategy to investigate the cellular target of AB in MDA-MB-231 cells. In total, 649 proteins were detected, among which 6 candidate AB-binding proteins were identified through the intersection of intracellular and extracellular DARTS (Figure S12A). Cellular thermal shift assay (CETSA) and DARTS assays were next used to verify the combing of AB with other proteins selected from DARTS-MS with significant differences, including CORO1A, VDAC, and PICH. CETSA results confirmed that AB destabilized CORO1A at 46 °C and 49 °C (Figure 2F, H), while AB had no effects on PICH (Figure S12C). Given the slight destabilization of VDAC was observed (Figure S12B), we immediately examined the impact of AB on the protein stability of CORO1A and VDAC through DARTS-WB assays. It was found that AB (50 µM) induced the destabilization of CORO1A during the proteolysis (Figure 2G, I) without affecting VDAC (Figure S12D), indicating that AB is directly bound to CORO1A. The microscale thermophoresis (MST) assay was used to measure the direct binding between AB and CORO1A, and a K_d_ value of 352 nM was obtained (Figure 2J). We performed a molecular docking analysis to understand the possible molecular binding pattern between AB and CORO1A. Three critical amino acid residues, including Arg225, Asp86, and Tyr180, played essential roles in the binding of AB with CORO1A (Figure 2K). Then, we generated CORO1A plasmids with the mutation in the above three critical amino acid residues. Further MST assays showed that the binding affinity of AB and mutant CORO1A tended to be significantly decreased with the K_d_ value of 248 μM (Figure 2L). Strikingly, CORO1A knockdown significantly alleviated the inhibition effect of AB on MDA-MB-231 cells (Figure S13A). Clonal formation assays showed that the inhibitory effect of AB on the proliferation of MDA-MB-231 cells can be reduced by CORO1A-WT plasmid supplementation, but the mutant CORO1A had no such effects (Figure S13B-C). Together, these data supported the critical role of CORO1A in AB-induced antitumor activity.

### CORO1A undergone neddylation-dependent degradation

**Figure 3:**
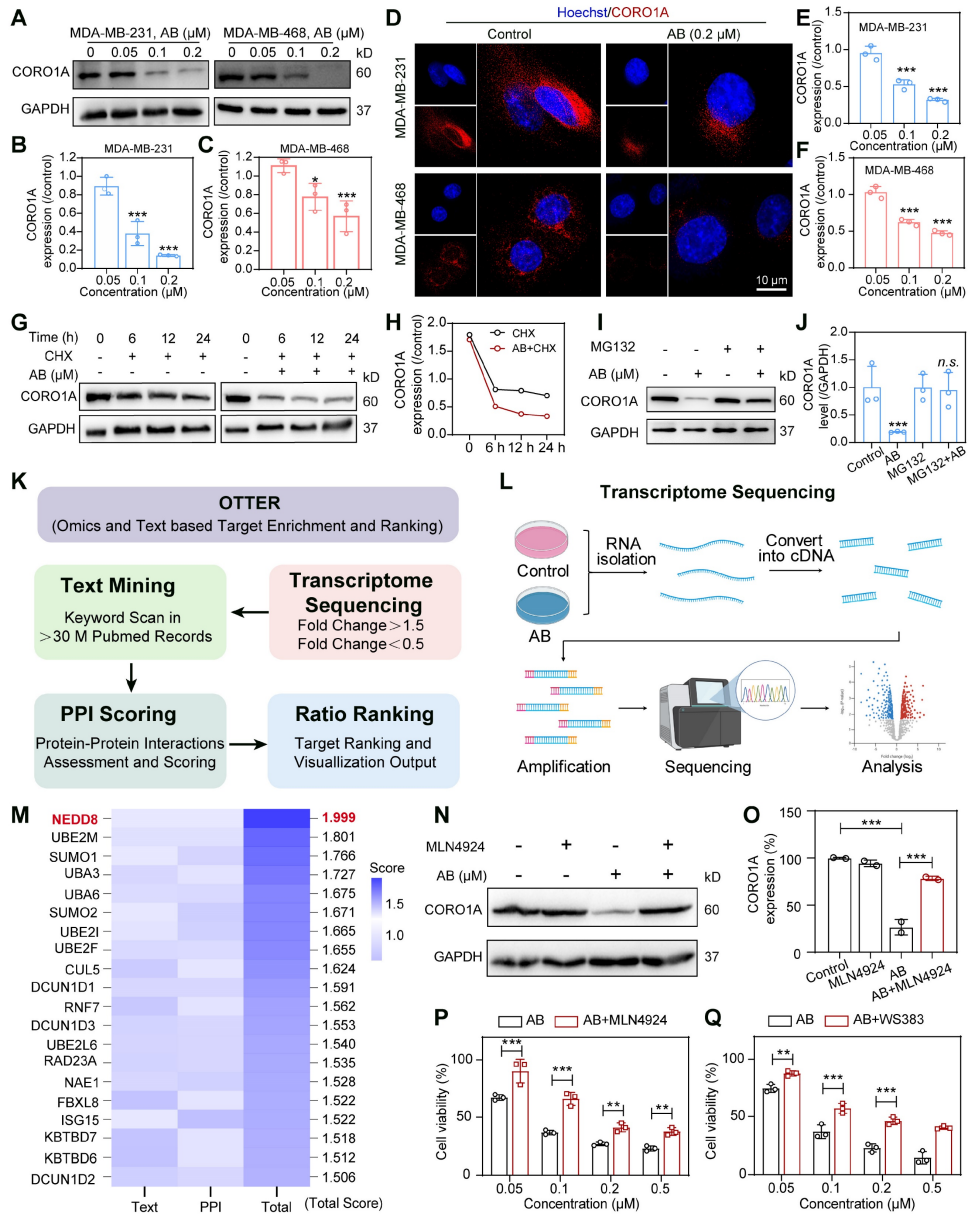
CORO1A underwent neddylation-dependent degradation induced by AB. **(A)** AB inhibited the protein expression of CORO1A in MDA-MB-231 and MDA-MB-468 cells. The analysis was showed in **(B)** and **(C)**. **(D)** The representative microscopic photographs of the CORO1A expression in MDA-MB-231 and MDA-MB-468 cells after AB (0.2 μM) treatment for 24 h. The analysis was shown in **(E)** and **(F)**. **(G)** AB accelerated the degradation of CORO1A in MDA-MB-231 cells. The analysis was showed in** (H)**. **(I)** MG132 (0.5 μM) rescued the degradation of CORO1A protein induced by AB (0.2 μM) in MDA-MB-231 cells. The analysis was shown in** (J)**. **(K)** The pattern diagram of OTTER analysis. **(L)** The pattern diagram of transcriptome sequencing.** (M)** The top 20 ubiquitin-related proteins were obtained using OTTER enrichment analysis. **(N)** MLN4924 reversed the degradation of CORO1A.** (O)** Analysis of **(N)**. **(P)** MLN4924 attenuated the inhibitory effect of AB on MDA-MB-231 cells. **(Q)** WS383 attenuated the inhibitory effect of AB on MDA-MB-231 cells. Data were presented as mean ± SD, *P ˂ 0.05, ***P ˂ 0.001 versus the control group.

To determine whether AB affected the cellular protein expression of CORO1A, we treated MDA-MB-231 and MDA-MB-468 cells with different concentrations of AB for 24 h. Compared with untreated cells, CORO1A protein levels were significantly decreased by AB in a dose-dependent manner (Figure 3A-C). While Quantitative Real-time PCR (qPCR) assays showed that AB (12 h or 24 h treatment) had no significant effect on the mRNA level of *CORO1A* (Figure S14). The immunofluorescence-based assay also revealed that AB (0.2 μM) induced CORO1A protein degradation (Figure 3D-F). We next validated the specific mechanism of AB degrading CORO1A using the cycloheximide (CHX) chase assay, which demonstrated that the combination of CHX and AB promoted the degradation rate of CORO1A (Figure 3G-H). The proteasomal inhibiter, MG132, was further utilized to detect the relationship between CORO1A degradation and the proteasome. In the presence of MG132, the AB-induced CORO1A degradation in MDA-MB-231 cells was markedly reversed (Figure 3I-J), while the autophagy inhibitor, CQ, had no significant effect on AB-induced degradation of CORO1A (Figure S15), supporting the proteasomal degradation as a cause for CORO1A depletion. To explore the mechanism of AB-induced ubiquitination and degradation of CORO1A, we further employed the Omics and Text-based Target Enrichment and Ranking (OTTER) tools that our research group co-developed in 2023 22, to analyze the interaction between all differentially expressed genes (Figure 3K-L). OTTER firstly conducts text mining of each differentially expressed genes (obtained by transcriptome sequencing results) in the PubMed abstract to calculate the text score of the gene. OTTER also considers the PPIs between these tops differentially expressed genes. The more PPI observed for a gene, the higher the PPI score calculated for that gene. Finally, all differentially expressed genes were ranked, and the top 20 candidate genes were selected for subsequent in-depth study according to the total scores, among which neural precursor cell-expressed developmentally downregulated 8 (*NEDD8*) showed the most vital interaction with *CORO1A* and ubiquitination (Figure 3M). The neddylation is the process of posttranslational protein modification by conjugating the ubiquitin-like protein, NEDD8, to target proteins and promote the activation of RING E3 complexes 23. Given the critical role of NEDD8 in the ubiquitination process of substrates, we therefore tested whether NEDD8 altered the protein expression of CORO1A. Surprisingly, in the presence of the inhibitor for the NEDD8-activating enzyme, MLN4924, the degradation of CORO1A (Figure 3N-O) was obviously attenuated, indicating the crucial role of the NEDD8 in the process of neddylation of CORO1A. Moreover, the anti-tumor effect induced by AB was attenuated in the presence of MLN4924 (Figure 3P). It has been reported that the interaction between DCN1 and UBC12 facilitates neddylation process, and the interruption of which leads to defective neddylation 24. WS383, inhibitor of DCN1-UBC12 binding, was further used to investigate the crucial role of the neddylation in AB efficacy. The result showed that WS383 exerted a similar attenuated effect on AB efficacy (Figure 3Q), suggesting an essential role in the NEDD8-mediated degradation of CORO1A.

### Identification of AB as a molecular glue degrader for CORO1A

**Figure 4:**
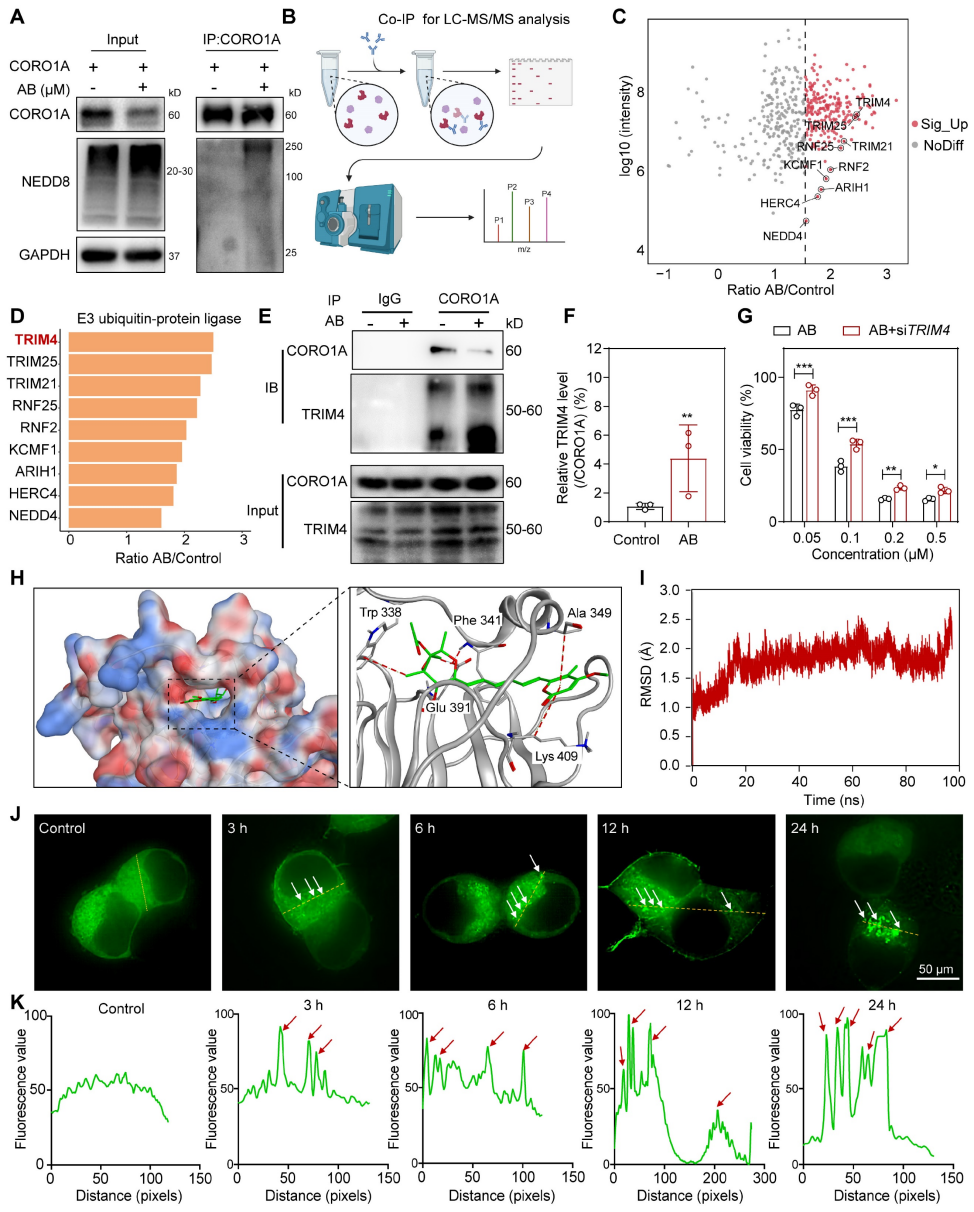
Identification of AB as a molecular glue degrader for CORO1A. **(A)** Co-IP assay using non-denaturing conditions confirmed the neddylation of CORO1A. **(B)** The pattern diagram of immunoprecipitation (IP) for LC-MS/MS analysis. **(C)** The proteins that interacted with CORO1A were identified by combining Co-IP and mass spectrometry assay.** (D)** TRIM4 changed remarkably upon AB treatment. **(E)** Co-IP assay in 293T cells confirmed the increased binding of TRIM4 to CORO1A after AB treatment. **(F)** Analysis of the relative TRIM4 level binding with CORO1A after AB treatment in IP group. **(G)** Knockdown of *TRIM4* attenuated the inhibitory effect of AB on MDA-MB-231 cells. **(H)** Molecular docking studies suggested that AB could bind to multiple sites of TRIM4 with a docking score of -8.26. **(I)** Molecular dynamics simulations showed that AB could stabilize the binding of CORO1A to TRIM4. **(J)** SPPIER assay visualized ternary complex formation. The analysis was conducted in **(K)**. Data were presented as mean ± SD, *P ˂ 0.05, **P ˂ 0.01, ***P ˂ 0.001 versus the group of AB treatment.

To test whether the reduced amounts of CORO1A were due to the increased NEDD8 modification induced by AB, HEK293T cells were transfected with CORO1A, and co-immunoprecipitation (Co-IP) assays were subsequently performed. Figure 4A showed that AB promoted the interaction between CORO1A and NEDD8. As previously reported, lysine 233 was the main neddylation site in CORO1A 25.

Then, we mutated (K to R) K233 in CORO1A (CORO1A-K233R) and performed the Co-IP assays. The results showed that the mutation of CORO1A (CORO1A-K233R) obviously diminished CORO1A neddylation in cells, indicating that K233 was the important neddylation site in CORO1A (Figure S16). To further identify which specific E3 ligase targets CORO1A for neddylation and subsequent proteasomal degradation, whole protein extracts of MDA-MB-231 cells with anti-CORO1A incubation were subjected to immunoprecipitation (IP) and determined by LC-MS/MS (Figure 4B). Nine proteins were associated with the ubiquitin-proteasome pathway and E3 ubiquitin-protein ligase (Figure 4C). A total of 853 proteins were identified from the UniProt database, and proteins associated with ubiquitin-proteasome pathway were received much attention according to the methods in the literature 26. Mainly, TRIM4 changed remarkably upon AB treatment, which might be a potential mechanism mediating CORO1A degradation (Figure 4D). Since TRIM4 has been revealed as a critical E3 ligase in CORO1A neddylation 25, we evaluated the interactions between CORO1A and TRIM4. It was shown that there was an interaction between CORO1A and TRIM4. AB treatment could reduce the level of CORO1A while significantly increasing the level of TRIM4 (Figure 4E-F). To evaluate the effects of TRIM4 in AB's inhibitory effects, we then performed transient transfection of siRNAs (siTRIM4) to knock down TRIM4 (Figure S17). For siTRIM4 cells, a mild inhibitory impact of AB was detected compared with the control group (Figure 4G). Molecular docking studies suggested that AB could bind to multiple sites of TRIM4 with a docking score of -8.26 (Figure 4H). To deeply explore the mechanism and function of AB in the promoted interaction between CORO1A and TRIM4, we speculated that AB might serve as a molecular glue to stabilize their binding. Consistent with this, molecular dynamics simulations also showed that AB could stabilize the binding of CORO1A to TRIM4 (Figure 4I). The separation of phases—based protein interaction reporter (SPPIER) method is widely used to detect robust and dynamic visualization of PPIs in living cells 27. We next performed SPPIER to visualize the ternary complex of CORO1A-AB-TRIM4 during the process of CORO1A degradation induced by AB. When the plasmids of TRIM4-EGFP-HOTag6 and CORO1A-EGFP-HOTag3 were transfected into HEK 293T cells for 24 h, AB (0.2 μM) was added for real-time detection. Consistent with the confirmed interaction between TRIM4 and CORO1A, we observed the homogeneous fluorescent droplet formation at 3 h after AB treatment, which was undetectable in the control group, indicating the formation of CORO1A-AB-TRIM4 trimer complex (Figure 4J-K). These observations suggested that CORO1A underwent neddylated degradation via TRIM4 after AB treatment, contributing to the inhibitory effect on TNBC cells.

### CORO1A degradation regulated TGF-β-Smads signaling and sequent cell apoptosis

**Figure 5:**
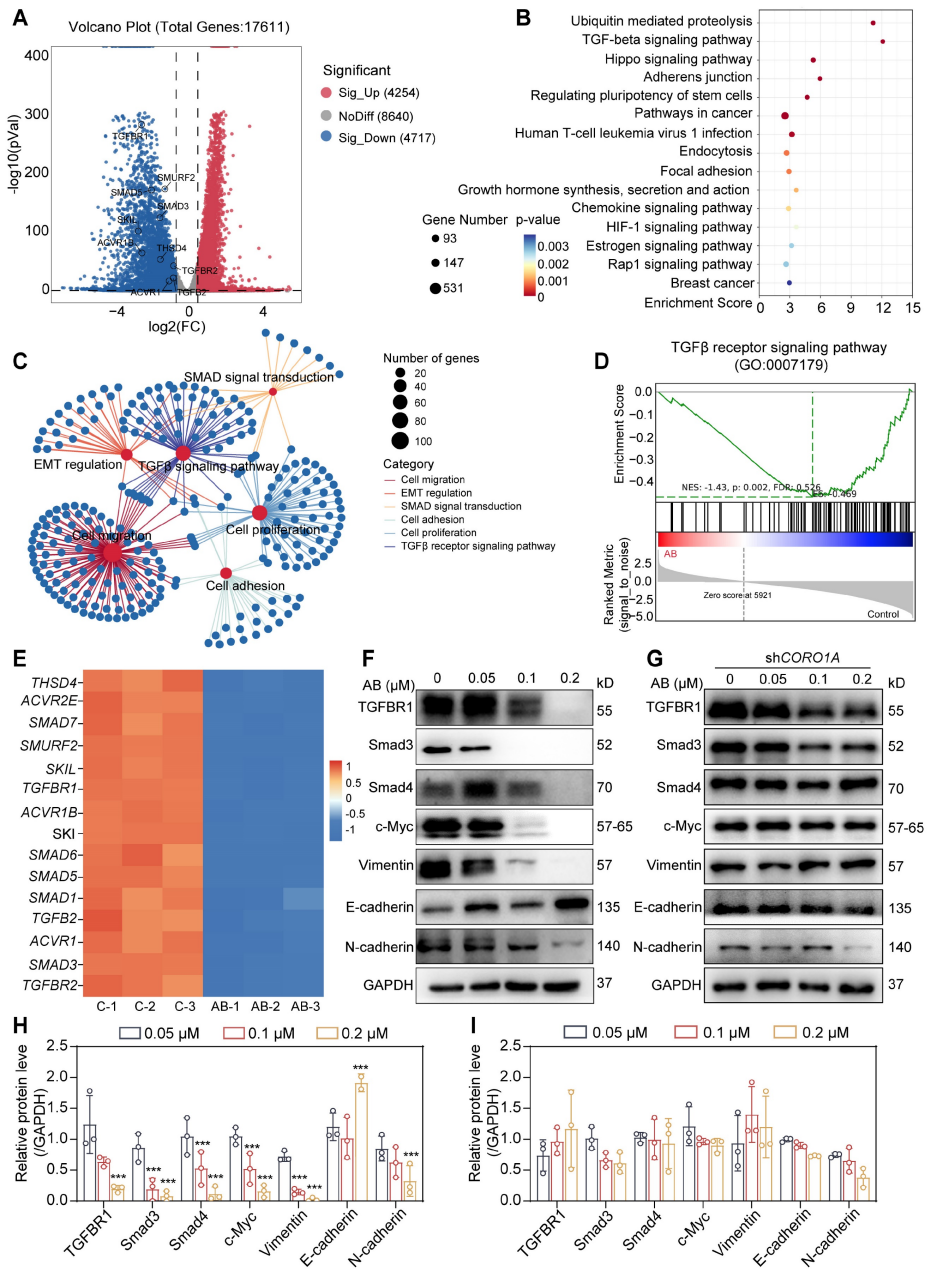
CORO1A degradation regulated TGFβ-Smads signaling and cell apoptosis. **(A)** The volcano plot represents differentially expressed genes. **(B)** KEGG enrichment analysis of quantitative proteomics.** (C)** Network of pathways that down-regulated with AB.** (D)** GSEA analysis showed a significantly decreased TGF-β signaling pathway induced by AB. **(E)** The heatmap analysis of down-regulated genes. **(F)** Western blot assay proved that AB downregulated TGFβ-Smads signaling. **(G)** Knockdown of *CORO1A* affected TGFβ-Smads signaling. **(H)** Analysis of** (F)**. **(I)** Analysis of **(G)**. Data were presented as mean ± SD, ***P ˂ 0.001 versus the control group.

A quantitative proteomics analysis was carried out to determine the mechanism of action by AB and detect the associated signaling pathways. In total, 8971 genes of differential expression with AB treatment were detected (Figure 5A), and pathway enrichment analysis was further performed as follows. The enrichment analysis of differential genes and signaling pathways showed that AB significantly affected the TGF-β and ubiquitin-mediated proteolysis pathway (Figure 5B). In addition, a network of pathways analysis with down-regulated genes showed that AB significantly down-regulated cell biological processes associated with tumor growth, including the TGF-β-Smads signaling pathway, cell migration, cell adhesion, cell proliferation, EMT, etc. (Figure 5C). Consistent with previous reports, TGF-β-induced changes in signaling pathways play an essential role in tumor progression. Gene set enrichment analysis (GSEA) further showed the significantly decreased TGF-β signaling pathway induced by AB (Figure 5D). Previous research also demonstrated that CORO1A could regulate TGF-β-Smads signaling in Th17 cells, and the knockdown of CORO1A resulted in a following decrease in the expression of Smad3 28. Therefore, we hypothesized that AB-induced CORO1A degradation led to TGF-β-Smad3 signaling pathway inhibition. To test this hypothesis, we first evaluated the involvement of CORO1A and AB in TGF-β-Smads signaling in TNBC cells. The results showed that AB attenuated protein expression of TGF-β, Smad3, and Smad4 (Figure 5E-F). TGF-β-induced EMT is crucially involved in the growth and metastasis of TNBC 29.

Literature also reported that c-Myc was a key component in TGF-β/Smads pathways and EMT process 30. In addition, when we performed the transcriptome sequencing assay when *CORO1A* was knocked down using siCORO1A, we also found that the expression of key genes in the TGF-β/Smads and EMT pathways was significantly reduced, indicating that *CORO1A* played roles in EMT and TGF-β signaling pathway (Figure S6D). So, we next detected CORO1A-related genes, including *Vimentin*, *N-cadherin*, and *c-Myc* (Figure 5F, H). To elucidate the effects of AB on TGF-β-Smads signaling pathways through CORO1A, we knocked down *CORO1A* using shRNA and examined the expression of crucial proteins in TGF-β signaling in TNBC cells. Knockdown of *CORO1A* led to attenuated inhibition effects on TGF-β-Smads signaling (Figure 5G, I). Moreover, the knockdown of *TRIM4* also induced a decrease in the content of related proteins in TGF-β signaling pathways (Figure S18). As the second messenger of cell death signal transduction, the calcium ion was closely related to mitochondrial function and ROS content 31. An elevated level of ROS damages cell membranes, resulting in intracellular calcium redistribution and extracellular calcium ion flow, thereby inducing tumor cell apoptosis 32. As mentioned above, AB significantly decreased the mitochondrial membrane potential of MDA-MB-231 cells (Figure S19A). At this point, the calcium ion concentration of MDA-MB-231 cells was increased rapidly (Figure S19B). *CORO1A* knockdown exhibited alleviated cell motility (Figure S20A-B) and migration ability (Figure S20C-D). In addition, the production of ROS and calcium ions induced by AB was significantly reversed (Figure S20E-K). Collectively, we proposed that AB might function as a molecular glue that induced CORO1A degradation via recruitment of the E3 ligase TRIM4, resulting in the neddylation of CORO1A and down-regulation of TGF-β-Smads signaling pathways to inhibit TNBC growth.

### Cell-derived xenograft tumor models and PDO models predicted the potential of molecular glues targeting CORO1A for TNBC therapy

**Figure 6:**
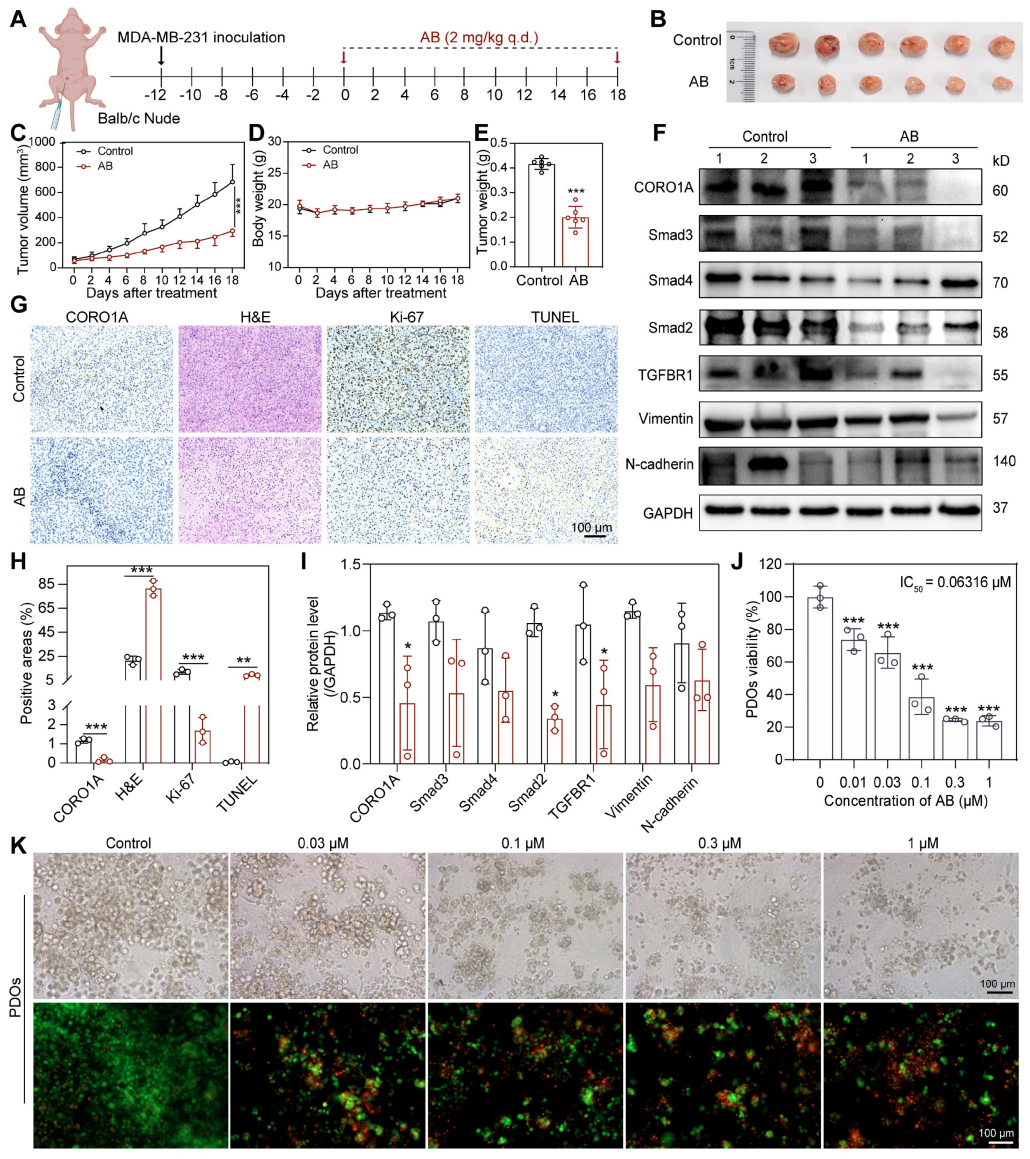
AB showed potent antitumor effects in TNBC-derived CDX models and PDO models. **(A)** Schematic diagram of *in vivo* study. **(B)** When mice were sacrificed, the tumors were photographed (n = 6 mice). **(C)** Tumor volume.** (D)** Body weight. **(E)** Tumor weight. **(F)** Western blot assay to detect the protein expression in tumor tissues.** (G)** IHC staining showed that AB induced increased necrosis and apoptosis, decreased proliferation, and decreased CORO1A expression.** (H)** Analysis of **(G)**. **(I)** Analysis of** (F)**. **(J)** AB inhibited TNBC PDOs formation with an IC_50_ value of 63.16 nM. (K) Representative images of Live/Dead staining of PDOs. Data were presented as mean ± SD, *P ˂ 0.05, **P ˂ 0.01, ***P ˂ 0.001 versus the control group.

To evaluate the antitumor effects of AB in TNBC cells, we next established the MDA-MB-231-derived xenograft tumor models (Figure 6A). Following intraperitoneal injection of AB (2 mg/kg) once daily for 18 days, we found that AB significantly decreased the tumor volume and tumor weight of mice compared with the control group without any obvious side effects (Figure 6B-E). IHC staining showed that AB induced an increased necrosis and apoptosis proportion of xenograft tumor tissues, a decreased proliferation ability of tumor cells, and reduced CORO1A protein expression (Figure 6G-H). At the same time, the blood routine and IHC staining of normal tissues showed no obvious side effects after AB treatment (Figure S21A-I). To test whether AB could induce the protein degradation of CORO1A *in vivo*, the levels of CORO1A and related proteins in the downstream signaling pathway in the tumor samples were evaluated by western blot assays. Protein expression of CORO1A and TGF-β-Smads signaling pathways were downregulated after AB administration (Figure 6F, I). These results indicated that AB served as a CORO1A degrader and exhibited potent inhibition effects of TNBC *in vivo*. Moreover, the antitumor effect of AB *in vivo* was significantly inhibited by MLN4924, suggesting the important role of neddylation induced by AB in inhibiting TNBC growth (Figure S22A-E). IHC staining showed that the decreased CORO1A protein expression and proliferation ability of tumor cells, and increased necrosis and apoptosis proportion of xenograft tumor tissues induced by AB were all weakened by MLN4924 (Figure S22F-H).

PDO models have now been developed for various cancers, providing a three-dimensional context, closer to the actual tumor microenvironment 33. Prior studies in TNBC PDOs have been proven to be suitable for preclinical drug screening and response prediction 33,34. To further investigate the translational value of AB, we then performed the PDO model of TNBC for predicting drug responses in clinics. PDOs were successfully constructed and treated with various concentrations of AB for consecutive 6 days. The results showed that AB could significantly inhibit organoid formation and survival with an IC_50_ value of 63.16 nM (Figure 6J). Subsequently, the LIVE/DEAD staining assay was further used to visualize the condition of organoids after AB treatment (Figure 6K). It was proved that AB could markedly decrease the number and viability of TNBC organoids even at the concentration of 0.03 µM (Figure 6K).

## Discussion

The Coronin family has long been recognized for its role in regulating actin dynamics, with aberrant expression linked to cancer development 6. Elevated CORO1A expression is associated with tumor migration and invasion, and preclinical evidence supports its potential as a marker for invasive breast cancer 7. In this study, we demonstrated the clinical significance of CORO1A in TNBC, showing that its knockdown reduces TNBC cell proliferation and tumorigenesis. However, the complex PPIs involving CORO1A present a challenge for developing effective inhibitors 14. Thus, novel therapeutic strategies targeting CORO1A are critically needed for TNBC treatment.

Molecular glue degraders have emerged as a promising approach for TPD strategies, allowing for the rapid degradation of targets in a substoichiometric manner 17. These degraders recruit target proteins to E3 ubiquitin ligases for ubiquitination and subsequent proteasomal degradation 35. Several molecular glues, such as thalidomide analogues and aryl sulfonamides, have demonstrated efficacy in treating various malignancies 36. Unlike classical inhibitors, which depend on specific target pockets, molecular glues expand the range of “druggable” proteins and offer favorable pharmacological properties 13, demonstrating the therapeutic potential of CORO1A-based molecular glues in TNBC. Molecular glues function by interacting with specific E3 ligase receptors. Tripartite motif (TRIM)-containing proteins, such as TRIM4, play a crucial role as E3 ubiquitin ligases 37. TRIM4, a member of the TRIM/RBCC protein family of RING E3 ligase, has been reported the ability to leverage the ubiquitin-proteasome pathway to degrade specific targeted proteins, and the low expression of which is highly associated with TNBC 38,39. Our findings indicate that knockdown of TRIM4 reduces the anti-tumor effects of Aurovertin B (AB), highlighting TRIM4's importance in TNBC treatment. Interestingly, AB retained its inhibitory effects at higher concentrations (0.5 and 1 μM) even after CORO1A or TRIM4 knockdown, suggesting that AB may target additional molecular pathways. Furthermore, AB has been shown to significantly upregulate dual-specificity phosphatase 1 40 or enhance the expression of NKG2D ligands on colorectal cancer cells, thereby sensitizing natural killer-mediated cancer immunotherapy 41. Therefore, we speculate that AB may interact with other proteins in cells to play antitumor roles. Future structural modifications of AB could enhance its specificity.

Neddylation, a post-translational modification like ubiquitination, plays a crucial role in protein degradation by conjugating NEDD8 to substrate proteins through a three-step enzymatic cascade 42. Studies have indicated that the BC-associated protein 3 (BCA3) suppressed NFκB-dependent transcription in a neddylation-dependent manner, thus exerting significant anti-tumor effects 20. However, it has also been reported that neddylation can switch PTEN from a tumor suppressor to a tumor promoter, thereby promoting BC tumorigenesis 21. To investigate neddylation's role in TNBC, we used the specific neddylation inhibitor MLN4924. Our data indicated that MLN4924 significantly inhibited AB's anti-tumor effects both* in vitro* and *in vivo*, underscoring the importance of the ubiquitination pathway mediated by TRIM4 and NEDD8 neddylation in TNBC progression.

Our study identifies AB as an effective antitumor agent that binds to CORO1A, promoting its interaction with NEDD8 and TRIM4. The biochemical activity of AB in mediating these interactions resembles that of other molecular glues, such as lenalidomide and indisulam 43,44. SPPIER assays, which detect PPIs through fluorophore phase transition, confirmed that AB facilitates the formation of the CORO1A-AB-TRIM4 complex, leading to CORO1A neddylation and proteasomal degradation 27. Molecular dynamics simulations further demonstrated that AB stabilizes CORO1A binding to TRIM4. CORO1A degradation notably suppressed the TGF-β-Smads signaling pathway and exhibited strong antitumor effects in TNBC PDO models. In addition to TGF-β and EMT signaling pathways, other related pathways were enriched following *CORO1A* knockdown, warranting further investigation. AB also inhibited cell migration and ROS production, with CORO1A knockdown alleviating these tumor phenotypes, indicating that AB induces TNBC inhibition in a CORO1A-dependent manner. Our findings establish AB as a natural CORO1A degrader, demonstrating its potential in targeting CORO1A through neddylation-mediated degradation and contributing to its antitumor effects.

## Methods

### Compounds and reagents

AB was provided by Prof. Zhan from Zhejiang University of Technology. Other compounds were purchased from Purification Technology (Chengdu, China). For *in vitro* experiments, AB was dissolved into DMSO (ST038, Beyotime) at a final stock concentration of 10 mM and stored at -20°C. Antibodies for CORO1A (#92904, 1:1000), Vimentin (#5714, 1:1000), E-cadherin (#3195, 1:1000), N-cadherin (#13116, 1:1000), GAPDH (#51332, 1:1000), HRP-conjugated anti-rabbit IgG (#7074, 1:5000), HRP-conjugated anti-mouse IgG (#7076, 1:5000), Alexa Fluor 488 conjugated-anti rabbit IgG (#4340, 1:1000) were purchased from Cell Signaling Technology. Antibodies for Smad3 (ab40854, 1:1000), p-Smad3 (ab52903, 1:1000), Smad4 (ab40759, 1:1000), Smad2 (ab40855, 1:1000), TGFBR1 (ab235578, 1:1000), c-Myc (ab32072, 1:1000) were obtained from Abcam. Antibody for TRIM4 (A15922, 1:1000) was purchased from Abclonal Technology. Antibodies of CORO1A (sc-100925, 1:50) and normal rat IgG (sc-2026, 1:200) for immunoprecipitation assay were all obtained from Santa Cruz Biotechnology.

### Cell culture

MDA-MB-231, MDA-MB-468, and HEK 293T cells were obtained from the Cell bank of the Shanghai Institute of Cell Biology, Chinese Academy of Sciences (SIBS, CAS). MDA-MB-231 and MDA-MB-468 cells were cultured in Leibovitz's L-15 (11415064, Gibco) medium. HEK 293T cells were cultured in DMEM (MA0212, Meilunbio) medium. All cultured mediums were supplemented with 10% fetal bovine serum (FBS, 10099141C, Gibco) and 1% penicillin/streptomycin (PWL062, Meilunbio). MDA-MB-231 and MDA-MB-468 cells were maintained in a humidified incubator at 37°C without CO_2_, and HEK 293T cells were cultured at 37°C containing 5% CO_2_.

### Bioinformatic analysis of CORO1A

Bioinformatic analysis, including gene expression and clinical prognosis, was performed using the online database (http://ualcan.path.uab.edu/ and Doi: https://kmplot.com/analysis/). R software GSVA package was used to analyze, choosing parameter as method = 'ssgsea'. RNA-sequencing expression (level 3) profiles and corresponding clinical information for CORO1A were downloaded from the TCGA dataset (Doi: https://portal.gdc.com). The correlations between CORO1A and pathway score were analyzed with Spearman by the database (Doi: https://www.aclbi.com/static/index.html#/).

### Virtual screening

Virtual screening was performed using Molecular Operating Environment 2020 software (MOE 2020). The 3D structure of CORO1A was downloaded from AlphaFold (Doi: https://alphafold.ebi.ac.uk/) and was energetically minimized by the QuickPrep Module in the MOE. The Site Finder methodology was used to predict the potential binding pocket of CORO1A. The target library containing 3194 compounds was read into MOE and converted into 3D structures. All compounds were prepared with the Wash Module to add H-atoms and Gasteiger charges. The candidates were selected for biological validation following flexible docking with force field refinement.

### Cell viability assay

Cell viability assay was determined by Cell Counting Kit-8 (MA0218, Meilunbio). MDA-MB-231 or MDA-MB-468 cells were seeded into 96-well plates (3599, Costar) at the density of 5×10^3^ cells/ well overnight. Drugs with different concentrations (0.05-1 µM) were added into the wells for 24 h. Then, the CCK-8 solution was added to the plate for 2 h, and the absorbance was measured at 450 nm using Cytation 5 (Biotek). Cell viability was calculated using the formula: cell viability = (OD_A_ - OD_B_)/ (OD_C_ - OD_B_) ×100%. A represented the group treated with drugs; B represented the blank group treated with the cell-cultured medium; C represented the control group.

### Calcein live/dead staining

MDA-MB-231 or MDA-MB-468 cells were seeded into 96-well plates at a density of 5×10^3^ cells/well overnight. AB with different concentrations (0.05-1 µM) was added to the wells for 24 h of incubation. 100 µL of AM/PI staining solution was added to each well for 10 min in the dark. Cells were washed twice with PBS, and the images were captured by the Operetta CLS High Content Analysis System (PerkinElmer). Calcein- AM (Ex/Em: 488/515 nm); PI (Ex/Em: 535/617 nm).

### Real-time cell analysis (RTCA)

Per the manufacturer's instructions, RTCA was performed using xCELLigence RTCA DP (Agilent). Briefly, the cultured medium (50 μL) was added to the E-Plate to test the cell baseline in the RTCA Station. The initial Cell Index of each well should be less than 0.063. Then, MDA-MB-231 or MDA-MB-468 cells were collected and seeded into each well at the density of 1×10^5^ cells/mL overnight. Finally, AB was added to the wells, and the Cell Index was collected for 48 h.

### Nanolive assay

MDA-MB-231 and MDA-MB-468 cells were grown overnight in confocal dishes (BS20GJM, BioSharp) at 5×10^4^ cells/dish density. Then AB (0.2 µM) was added to the dishes, and the images were captured by Nanolive 3D explorer.

### Colony formation

MDA-MB-231, MDA-MB-468, or MCF7 cells (1×10^3^ cells/well) were cultured into 12-well plates and treated with AB (0.05-1 µM) for 24 h. The cultured medium was changed every 3 days for 2 weeks. The colonies were fixed using paraformaldehyde and stained with crystal violet. Images were captured using Cytation 5 (Agilent Biotek).

### Transwell assay

The migration assay was performed in 24-well Transwell Boyden chambers with the 8.0 µm polycarbonate membrane (3422, Costar). The bottom chambers were filled with 600 µL medium containing 20% FBS. MDA-MB-231 or MDA-MB-468 cells (5×10^4^ cells/well) with AB (0.05-1 µM) treatment were suspended in 100 µL medium and seeded into the top chambers for 8 h. Migrated cells were fixed with paraformaldehyde and stained with crystal violet. The images were captured with a Leica microscope for analysis.

### Wound healing assay

MDA-MB-231 or MDA-MB-468 cells (2.5×10^5^ cells/well) were seeded into a 12-well plate (3513, Costar) and allowed to grow to confluence. After scratching with pipette tips, the cells were treated with different AB (0.05-1 µM) for 12 h. All the images were captured by microscope (Leica).

### ROS detection

MDA-MB-231 and MDA-MB-468 cells were seeded into the 96-well plate (5×10^3^ cells/ well) and 12-well plate (1.5×10^5^ cells/ well) overnight and treated with AB (0.05-0.5 µM) for 24 h. For cells in the 96-well plate, DCFH-DA (S0033M, Beyotime) was added to each well for 30 min. Then, the cells were washed twice with PBS and added with 100 µL DAPI (D9542, Sigma) for another 10 min. When the staining buffer was replaced with PBS, the images were captured by the Operetta CLS High Content Analysis System (PerkinElmer). ROS: Ex/Em: 488/515 nm; DAPI: Ex/Em: 535/617 nm. For the 12-well culture plate, cells were collected and stained with 300 µL DCFH-DA for 30 min. Flow cytometry (Beckman) assay was further performed for ROS detection.

### Apoptosis detection

MDA-MB-231 and MDA-MB-468 were seeded into the 12-well plate (1.5×10^5^ cells/ well) overnight and treated with AB (0.05-1 µM) for 24 h. Then, the cells were digested with trypsin (without EDTA) and washed with PBS three times. When cells were incubated with Annexin V for 15 min and stained with PI for 5 min, the flow cytometry (Beckman) was further performed to detect tumor cell apoptosis.

### Drug affinity responsive target stability (DARTS)

DARTS assay is a new technique based on the principle that when a small molecule compound binds to a protein, the enzymatic stability of the protein changes accordingly, which is universally applicable for drugs screening and target identification because it requires no modification of the drug and is independent of the mechanism of drug action 45-47. In this study, TNBC cells were seeded into 60 mm dishes overnight and lysed with NP-40 buffer (P0013F, Beyotime) containing phosphatase inhibitor (4906845001, Roche) as well as complete protease inhibitor (11697498001, Roche) at 4°C. Concentrations of proteins were quantified by BCA assay kit (P00091, Beyotime). Then, the cell lysates were treated with AB (50 µM) and an equal volume of DMSO for 1 h. Pronase with different proportions was added into the above proteins and incubated for 30 min at room temperature. Finally, the complete protease inhibitor was added to terminate the reaction, and cell lysates were denatured for Western blot detection.

### Cellular thermal shift assay (CETSA)

Cellular thermal shift analysis (CETSA) is a widely used biophysical technique introduced in 2013 and is now usually used to verify and quantify drug target conjugation in cells and tissues of different species 13,45,46. In this study, TNBC cells were seeded into 60 mm dishes overnight and treated with AB (50 µM) and an equal volume of DMSO for 3 h. Then, the cells were collected and lysed with NP-40 buffer (P0013F, Beyotime) containing phosphatase inhibitor (4906845001, Roche) and complete protease inhibitor (11697498001, Roche) at 4°C. When the proteins were quantified by BCA assay kit (P00091, Beyotime), the cell lysates were divided into equal volumes and heated from 37°C to 52°C for 3 min. After centrifugation, cell lysates were collected for western blot detection.

### Western blot assay

MDA-MB-231 and MDA-MB-468 cells (2.5×10^5^ cells/well) were grown into 6-well plates (3516, Costar) and treated with AB for 24 h. Cells were collected and lysed with NP-40 buffer containing phosphatase inhibitor and complete protease inhibitor at 4°C. Concentrations of proteins were quantified by BCA assay kit and denatured using SDS buffer (LT101S, Epizyme). 10% SDS-PAGE (PG112, Epizyme) was then used to separate critical proteins. When proteins were transferred to PVDF membranes (ISEQ00010, Millipore), the membrane was blocked for 2 h using 5% non-fat milk (1706404, Bio-Rad) and incubated with primary antibodies overnight. At room temperature, the membrane was incubated with HRP-conjugated antibodies for another 1 h. Enhanced chemiluminescence detection reagents (36208ES76, Yeasen) were added, and the ChemiDoc Imaging system (733BR5417, Bio-Rad) was used for protein detection.

### Molecular docking

MOE software was applied for molecular docking. The chemical structure of AB was downloaded from PubChem: Doi: https://pubchem.ncbi.nlm.nih.gov/. The protein structures of TRIM4 (PDB code: 2EGM) and CORO1A (PDB code: 2aq5) were downloaded from the PDB database: Doi: https://www.rcsb.org/.

### Microscale thermophoresis (MST) assay

HEK 293T cells overexpressed EGFP, or EGFP-tagged CORO1A, were lysed with NP-40 buffer containing phosphatase inhibitor and complete protease inhibitor at 4°C. The binding affinity of AB and CORO1A was detected following the manufacturer's protocol. Briefly, fluorescence signals of cell lysates were detected using Monolith NT.115 instrument (Nanotemper Technology) and diluted down to proper concentration. The ligand was diluted 15 times and mixed with an equal volume of cell lysate. The mixture solutions were absorbed by capillaries, and the MST signal of targets was monitored. The dose-response curve resulting from MST measurements was then analyzed using either a Kd-fit or Hill-fit model to determine the dissociation constant (Kd).

### siRNA transfection

MDA-MB-231, MDA-MB-468, or MCF7 cells (2.5×10^5^ cells/well) were grown into 6-well plates (3516, Costar) overnight. siRNA and transfect mate (Genepharma) were diluted with 200 µL Opti-MEM. Then, the above siRNA complex was mixed with the mate complex for 15 minutes. After 6 h transfection, the medium was replaced with fresh cultured medium. Western blot assay and CCK-8 assay were performed after 48 h transfection.

### Proteomics

MDA-MB-231 cells were grown into 60 mm dishes overnight and treated with AB (0.2 µM) for 24 h. Cells were collected and lysed with NP-40 buffer containing protease and phosphatase inhibitors. When the proteins were quantified using the BCA assay kit, a TMT-labelled quantitative proteome analysis was performed as previously described.

### Transcriptome sequencing

MDA-MB-231 cells were grown into 60 mm dishes overnight and treated with AB (0.2 µM) for 24 h or transfected with si*CORO1A* for 48 h. Cells were collected, and the total RNA was extracted using triazole. Then, the total transcriptome sequencing assay was carried out by OE Biotech Co., Ltd. (Shanghai, China).

### Co-Immunoprecipitation (Co-IP) assay

HEK 293T cells were grown in 100 mm dishes (430167, Corning) overnight and were transfected with CORO1A plasmid DNA and TRIM4 plasmid DNA (10 μg/dish) for 36 h. Cells were treated with AB (0.2 μM) for 24 h. Cells were collected and lysed in IP lysis buffer (P0013, Beyotime) containing protease and phosphatase inhibitors. Protein lysates were diluted to the concentration of 1 mg/mL. For 1 mg protein, 15 μL of anti-CORO1A (0.2 μg/μL) or an equal amount of rat IgG antibody (0.5 μg/μL) was added into cell lysates and incubated overnight at 4°C. 30 μL Protein A beads (Santa Cruz Biotechnology Inc., sc-2003) were added to bind antibodies and incubated for 2 h at 4°C. Beads were collected and washed 5 times with 200 μL IP lysis buffer. Finally, the protein samples were denatured with 3× SDS loading buffer for Western blot.

### Co-immunoprecipitation assay for LC-MS/MS analysis

MDA-MB-231 cells grown in 100 mm dishes (430167, Corning) were treated with AB (0.2 μM) for 24 h. Cells were lysed with IP lysis buffer (P0013, Beyotime) containing protease and phosphatase inhibitors. 500 μg proteins in the control group and AB group were incubated with anti-CORO1A (0.2 μg/μL) overnight at 4°C. After precipitating with Protein A beads, the precipitant was denatured with 3× SDS loading buffer for PAGE Gel electrophoresis. The gels with different bands were used for mass spectrometry analysis.

### Immunofluorescence

MDA-MB-231 cells and MDA-MB-468 cells (5×10^4^ cells/ dish) were grown in confocal dishes (BS20GJM, BioSharp) overnight and were treated with 0.2 µM AB for 24 h. Cells were washed with PBS for 3 times and fixed with paraformaldehyde (P0099, Beyotime) for 20 min and then permeabilized with 1% Triton X-100 (ST795, Beyotime) for another 5 min. Protein was blocked with 1% BSA for 1 h at room temperature and incubated with primary antibody for CORO1A (1:1000, 17760-1-AP, Proteintech) overnight at 4°C. Cells were incubated with Goat anti-Rabbit IgG-HRP (1:500, abs20040, Absin) conjugated secondary antibody for 2 h at room temperature and stained with Hoechst 33342 for 10 min. Images were captured by GE DeltaVision OMX SR.

### Separation of phases-based protein interaction reporter (SPPIER)

The SPPIER method is widely used to detect robust and dynamic visualization of PPIs in living cells 13,27,48. In this study, CORO1A-EGFP-HOTag3 plasmids (PPL03046-2a) and TRIM4-EGFP-HOTag6 (PPL03042-2b) plasmids were constructed and identified by Geneppl technology, co, Ltd (Nanjing, China). HEK 293T cells were grown in laser-confocal petri dishes. Cells were transfected with CORO1A-EGFP-HOTag3 plasmid and TRIM4-EGFP-HOTag6 using DNA transfection reagent (TF20121201, Neofect) and imaged after 36 h transfection. 0.2 µM of AB was added to dishes, and time-dependent images were captured by GE DeltaVision OMX SR.

### Xenograft animal model

Four-week-old female BALB/c nude mice were obtained from Shanghai Slake Experimental Animal Co., Ltd. and housed under specific pathogen-free conditions. Animal experiments were approved by the Ethical Committee of the Shanghai University of Traditional Chinese Medicine (PZSHUTCM2303030005). For* in vivo* studies, 5×10^6^ MDA-MB-231 cells in 100 µL serum-free culture medium were subcutaneously injected into mice's fourth breast pad. When the tumor volume reached 300 mm^3^, the tumor was resected, cut into 5 mm× 5 mm×5 mm pieces, and replanted into the fourth breast pad of 12 mice. When the average tumor volume reached 50 mm^3^, mice were randomly divided into two groups (6 mice per group) and injected intraperitoneally with or without AB (2 mg/kg/day). Tumor volume (Length and Width) and body weight were recorded once a day. Tumor volumes were calculated using the formula: tumor volume = length × width × width/2. When tumor volume reached 600-800 mm^3^, mice were sacrificed, and tumors were resected. To further investigate the anti-tumor effect of AB *in vivo*, tumor tissues were prepared for paraffin sections, immunohistochemical staining, and western blot assay. To identify the specific role of neddylation in TNBC, we reconstructed the animal model according to the above method and implanted the tumor. When the average tumor volume reached about 50 mm^3^, mice were randomly divided into four groups (6 mice per group): Model, AB (2 mg/kg, twice a day), MLN4924 (7.5 mg/kg/day), AB combination with MLN4924. The other experiments were consistent with the above method.

### Patients-derived organoid (PDO) model of TNBC

TNBC PDO models were established as previously described by Nanchang Royo Biotech Co, Ltd (Nanchang, China). Briefly, 100 organoids/well were laid into 96-well plates and incubated in 5% CO_2_/95% air at 37°C for 6 days. On day 3, the freshly prepared AB and medium mixture were added. At the end of the sixth day, 100 µL of AM/PI staining solution was added to each well for 10 min in the dark. Cells were washed twice with PBS, and the images were captured by Cytation 5 (Biotek). Calcein- AM (Ex/Em: 488 / 515 nm); PI (Ex/Em: 535 / 617 nm). The survival rate was calculated by the formula as follows: survival rate = (organoids alive on day 6 with AB treatment/organoid alive on day 0 with AB treatment) / (organoids alive on day 6 in control group/organoids alive on day 0 in the control group) × 100%.

### Three dimensional (3D) bioprinting

MDA-MB-231 spheroids were generated using the improved 3D bioprinting technique. Briefly, MDA-MB-231 cells (5×10^4^ cells / well) were mixed with 10 μL of 8% (w/v) GelMA containing 0.2% (w/v) lithium phenyl (2,4,6-trimethylbenzoyl) phosphinate (LAP). The mixture was loaded onto the printing stage and processed by a 3D printing system (Cyberiad Biotech). The bioprinted 3D spheroids were generated with a thickness of 10 μm and a diameter of 3 mm. After cultured at 37 °C with 5% CO2 for 24 h, the 3D spheroids were treated with different concentrations of AB for 48 h. The LIVE / DEAD assay was further performed according to the manufacturer's instructions and visualized by the Cytation 5 cell imaging multimode reader.

### Statistical analysis

All data were presented as mean ± SD from at least 3 independent experiments. Statistical analysis and graphical representation of the data were performed using GraphPad Prism 8.0 (GraphPad Software, San Diego, CA). Differences between groups were examined using one-way ANOVA or Two-way ANOVA.

## Supplementary Material

Supplementary figures and table.

## Abbreviations

AB: Aurovertin B
ACC: Adrenocortical carcinoma
BC: Breast cancer
BCA3: BC-associated protein 3
BRCA: Breast invasive carcinoma
BLCA: Bladder Urothelial Carcinoma
CETSA: cellular thermal shift assay
CHX: cycloheximide
CORO1A: Coronin1A
COAD: colon adenocarcinoma
DARTS: drug affinity response target stability
ER: estrogen receptor
HER2: epidermal growth factor receptor 2
IHC: immunohistochemical
IP: immunoprecipitation
LIHC: liver hepatocellular carcinoma
KICH: Kidney Chromophobe
MS: mass spectrometry
MST: microscale thermophoresis
NEDD8: neural precursor cell-expressed developmentally downregulated 8
OTTER: Omics and Text based Target Enrichment and Ranking
ROS: reactive oxygen species
PR: progesterone receptor
RT: radiotherapy
RTCA: real time cellular analysis
READ: Rectum adenocarcinoma
SPPIER: separation of phases-based protein interaction reporter
TNBC: triple-negative breast cancer
TPD: targeted protein degradation
