# Supplementary material for: TRIM4 enhances small-molecule-induced neddylated-degradation of CORO1A for triple negative breast cancer therapy
Source: Theranostics. 2024 Oct 21;14(18):7023–41. doi: 10.7150/thno.97662 (PMC11610137; doi:10.7150/thno.97662)
Supplement: Supplementary file 1 — Supplementary figures and table. [file thnov14p7023s1.pdf]

A

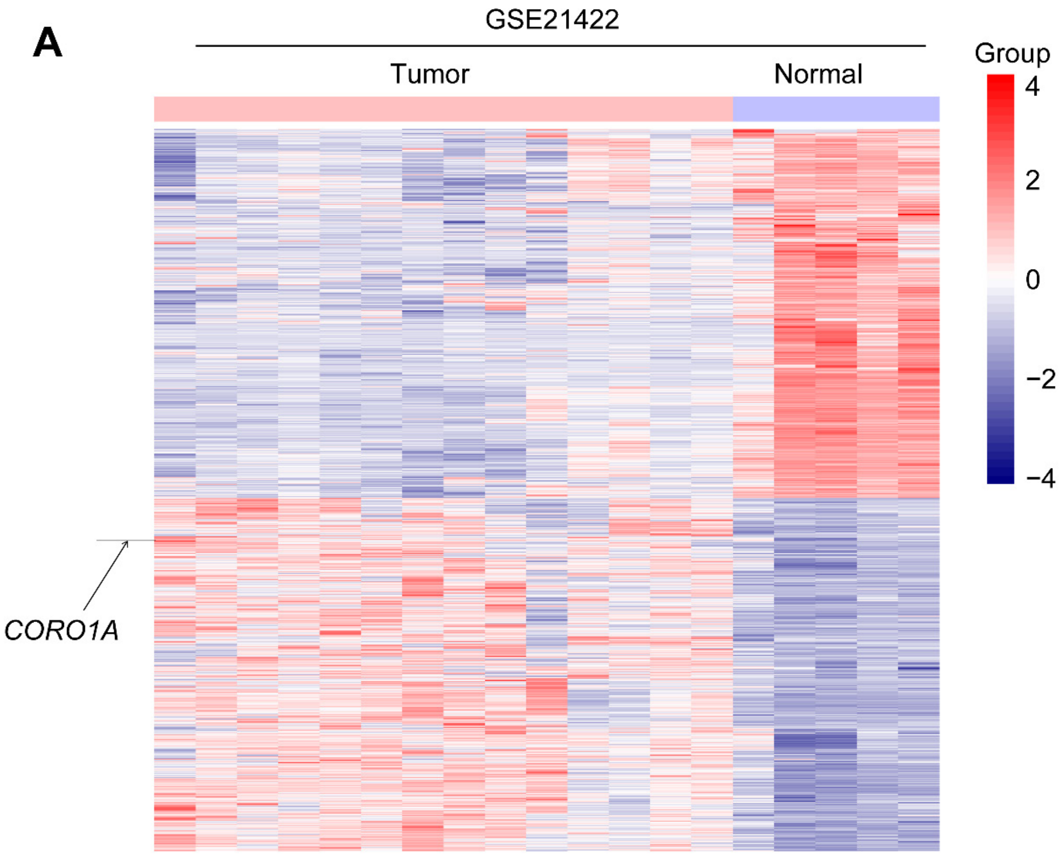

Figure S1. *CORO1A* overexpression in BC patients. **(A)** *CORO1A* expression was significantly increased in BC patients compared to the normal tissues using the GSE21422 database.

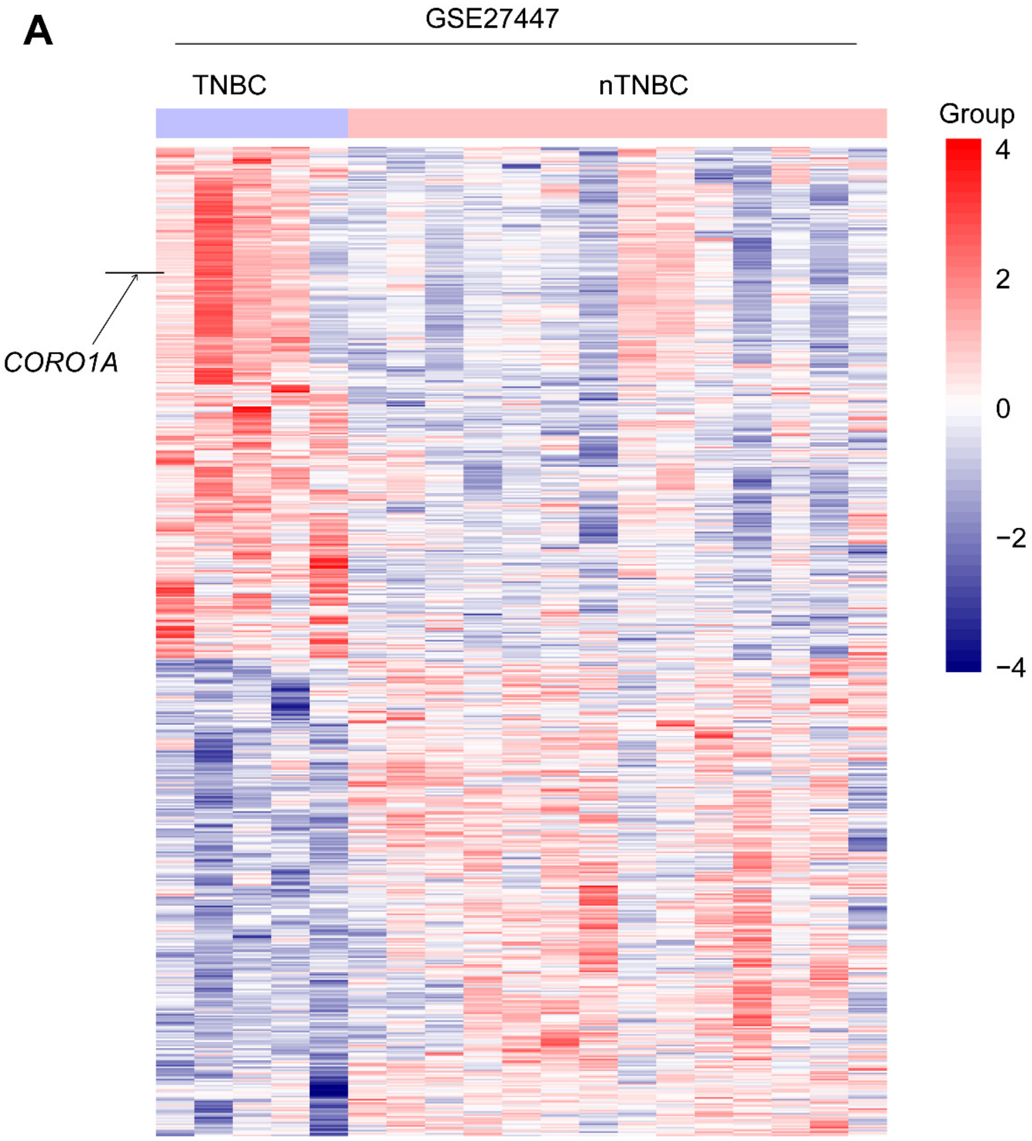

Figure S2. *CORO1A* overexpression in TNBC patients. **(A)** *CORO1A* expression was significantly increased in patients with TNBC (GSE27447 database) compared to non-TNBC (nTNBC) patients.

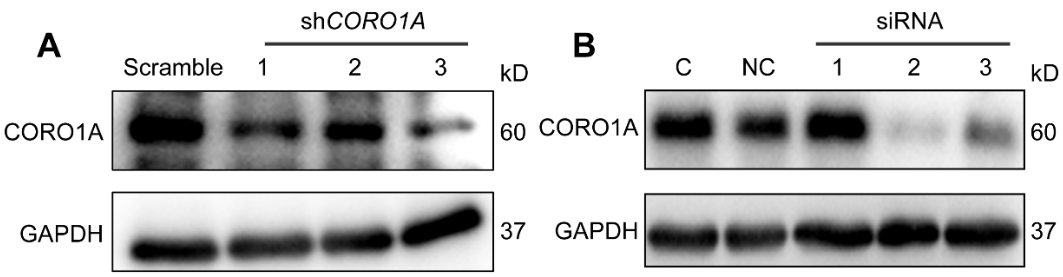

Figure S3. Protein expression of CORO1A. **(A)** Western blot assay determined the knockdown of *CORO1A* in MDA-MB-231 cells using lentiviral shRNA. **(B)** *CORO1A* was knocked down through three siRNAs (siRNA 1, 2, and 3) for 48 h in MDA-MB-468 cells.

NC represented negative control. The numbers 1,2, and 3 represented three different shRNA names or siRNA names, respectively.

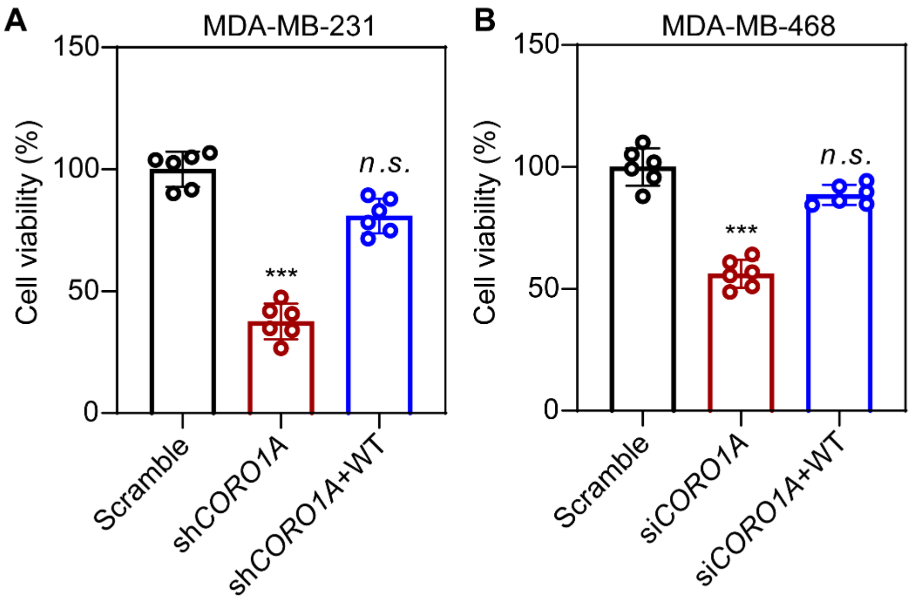

Figure S4. Cell viability of MDA-MB-231 and MDA-MB-468 cells. **(A)** The supplementation of CORO1A WT plasmid (WT) in the MDA-MB-231 sh*CORO1A* group restored cell vitality. **(B)** The supplementation of CORO1A WT plasmid (WT) in the MDA-MB-468 si*CORO1A* group restored cell vitality. The cell viability was detected using CCK-8 assay. Data were presented as mean ± SD, ***$P < 0.001$, *n.s.* indicated no statistical significance.

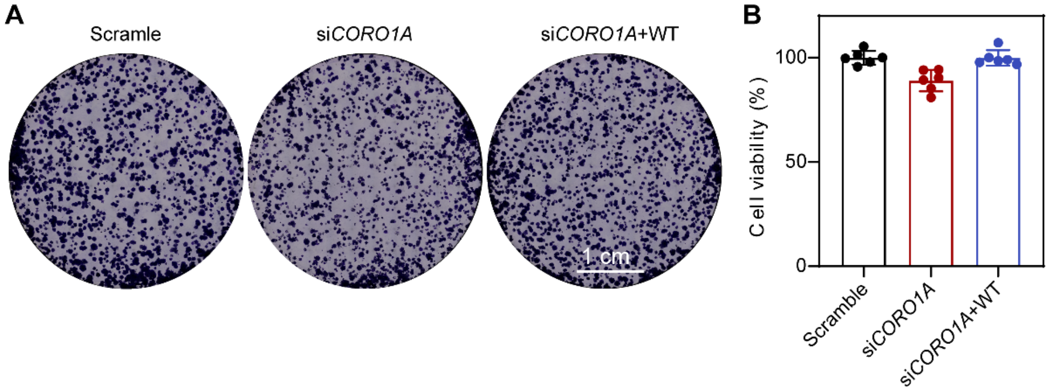

Figure S5. Representative images of colony formation and analysis in MCF7 cells. **(A)** Knock down of *CORO1A* using siRNA-2 showed no significant effects on MCF7 proliferation. In addition, the supplementation of CORO1A in the MCF7 si*CORO1A* group also had no significant effect on cell proliferation. (B) The cell viability was detected using CCK-8 assay.

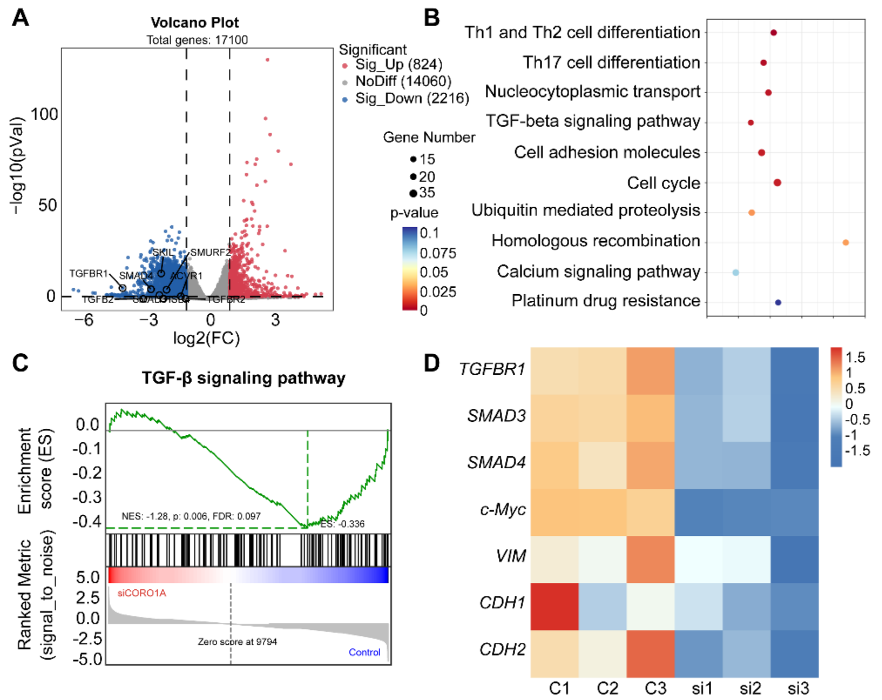

Figure S6. Knock down of *CORO1A* regulated TGFβ-Smads and EMT signaling pathways. (A) The volcano plot represented differentially expressed genes. **(B)** KEGG enrichment analysis of differentially expressed genes. **(C)** GSEA analysis showed a significantly decreased TGF-β signaling pathway. **(D)** The heatmap analysis of down-regulated genes.

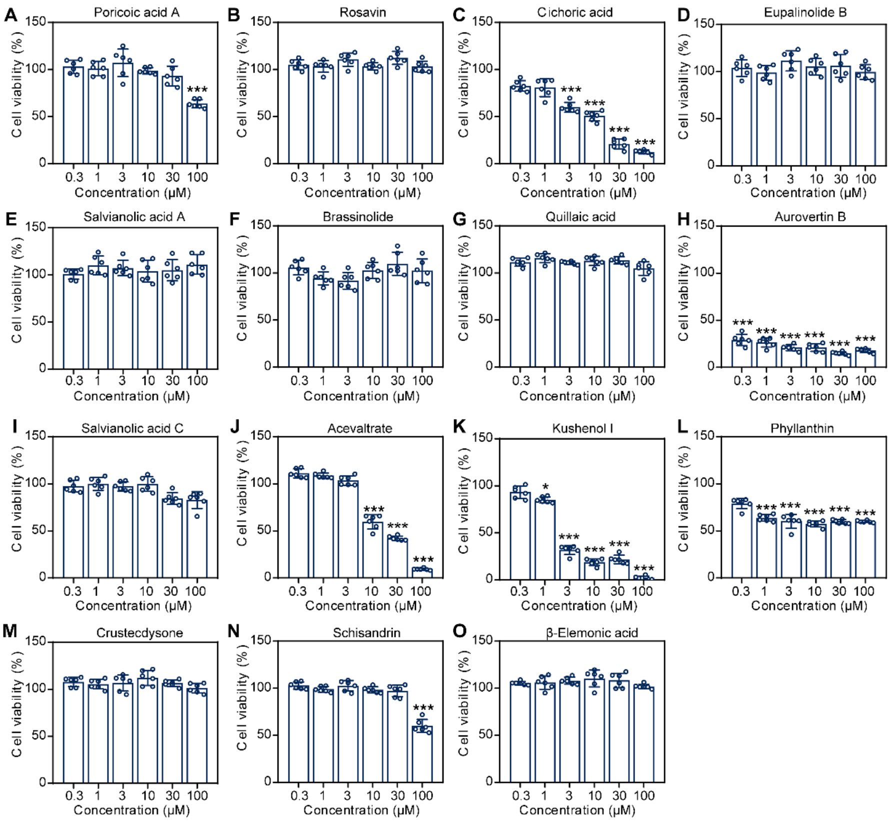

Figure S7. Cell viability detection. Cell viability of MDA-MB-231 was detected using CCK-8 assays after Poricoic acid A **(A)**, Rosavin **(B)**, Cichoric Acid **(C)**, Eupalinolide B **(D)**,

Salvianolic acid A **(E)**, Brassinolide **(F)**, Quillaic acid **(G)**, AB **(H)**, Salvianolic acid C **(I)**, Acevaltrate **(J)**, Kushenol I **(K)**, Phyllanthin **(L)**, Crustecdysone **(M)**, Schisandrin **(N)**, or β-Elemonic acid **(O)** treatment for 24 hours. Data were presented as mean ± SD, ****P* < 0.001.

Cell lines	IC_{50} (μM)	Cell lines	IC_{50} (μM)
MDA-MB-231 (TNBC)	< 0.1	Hs578T (TNBC)	>100
MDA-MB-453 (TNBC)	≈ 0.1	4T1 (BC)	>10
MDA-MB-468 (TNBC)	≈ 0.1	RKO (CRC)	>10
BT549 (TNBC)	>100	DLD-1 (CRC)	>100
HCC1937 (TNBC)	>10	HCT116 (CRC)	>100
MDA-MB-436 (TNBC)	>100	ASPC-1 (PAAD)	>100
MCF7 (BC)	>10	MCF-10A (Normal cells)	>100

Figure S8. Cell viability after AB treatment. Cell viability of different cancer types was detected using CCK-8 assays after AB treatment for 24 hours.

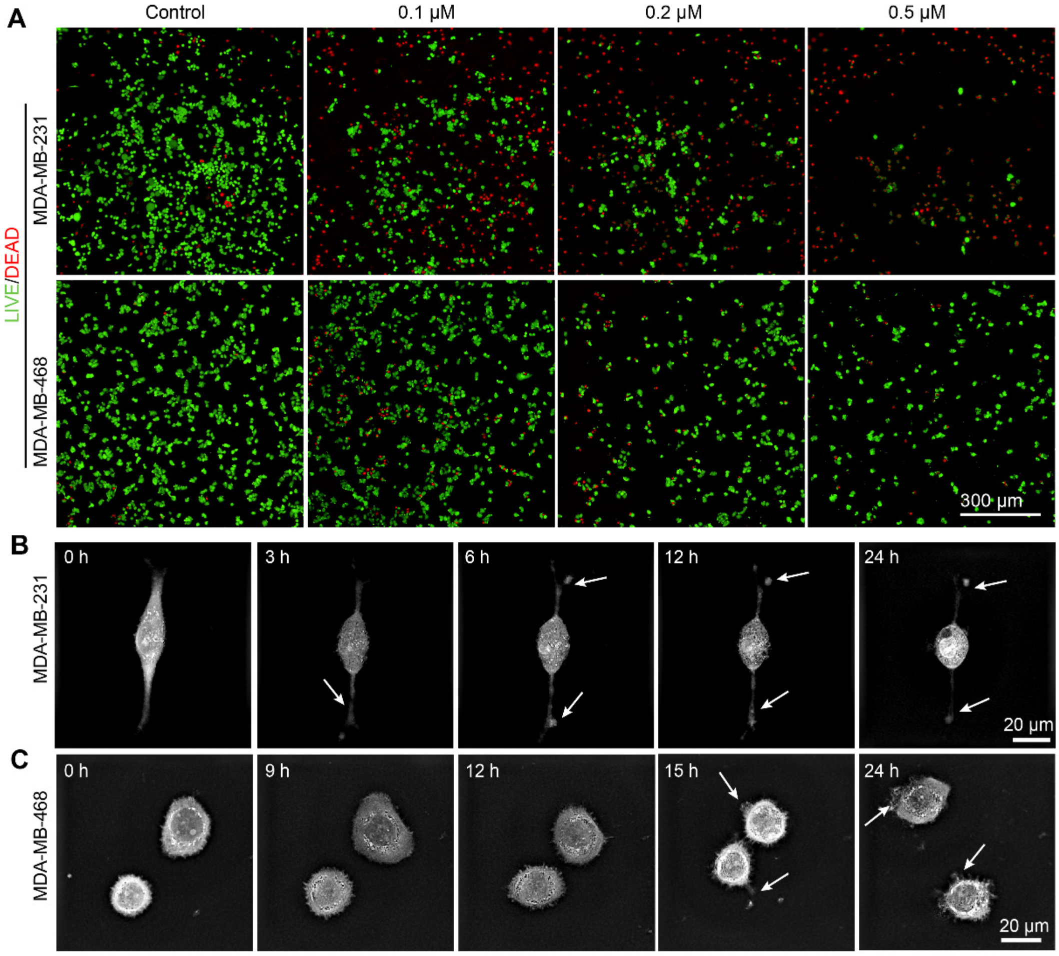

Figure S9. AB inhibited cell viability. **(A)** Representative images of LIVE/DEAD staining in MDA-MB-231 and MDA-MB-468 cells after AB treatment for 24 h. Nanolive assays were

further showed that AB rapidly induced the loss of cell morphology and the rupture of contents in MDA-MB-231 **(B)** and MDA-MB-468 cells **(C)**.

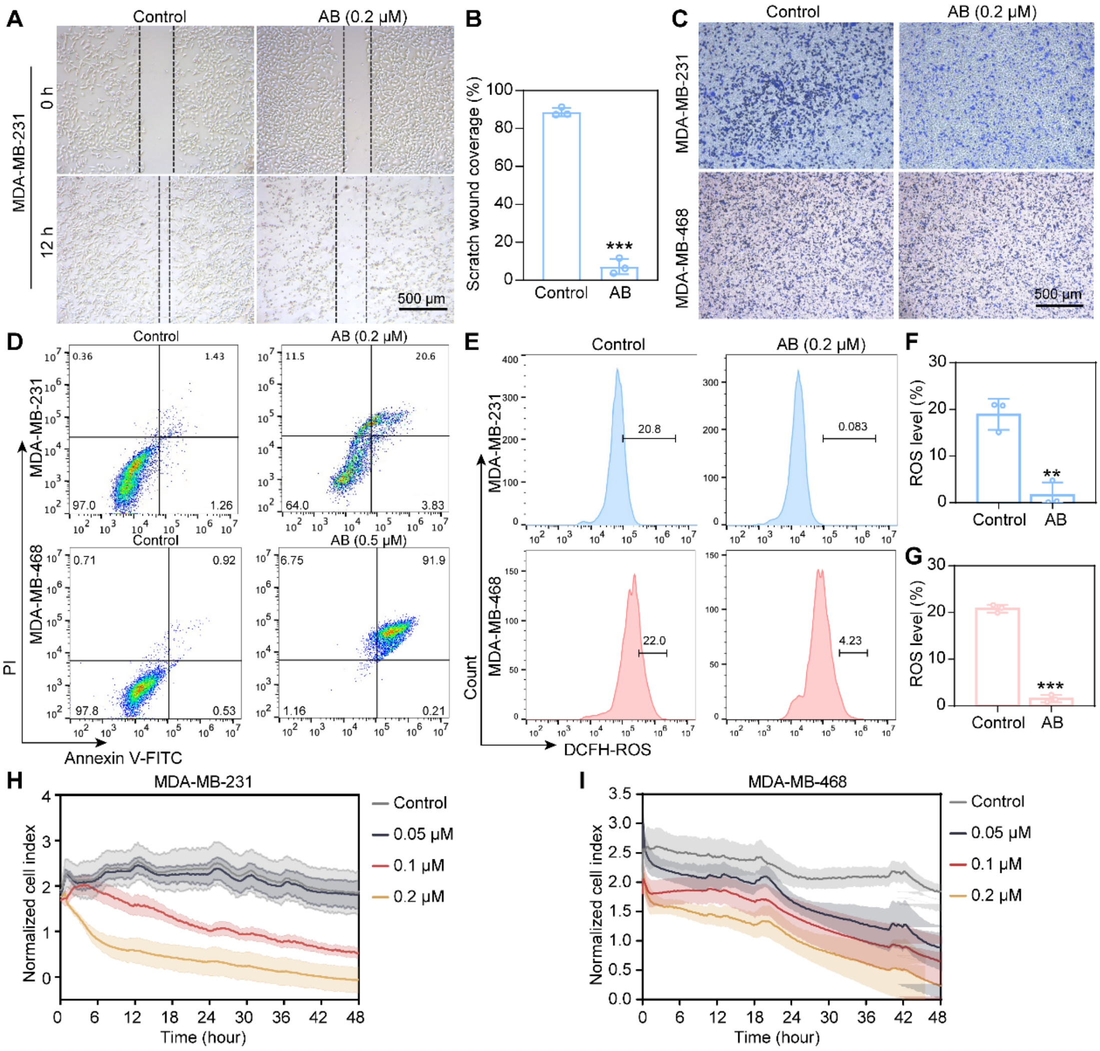

Figure S10. AB exerted antitumor effects on TNBC cells. **(A)** The wound healing assay showed that AB induced the inhibition of cell motility. The analysis was showed in **(B). (C)** Transwell assay showed that AB inhibited the cell migration. And the analysis was showed in Figure 2C. Flow cytometry assay also showed that AB induced cell apoptosis **(D)** and inhibited ROS production **(E)**. The analysis of cell apoptosis was showed in Figure 2D. The analysis of ROS production in MDA-MB-231 and MDA-MB-468 cells was showed in (F) and (G). RTCA assay showed that AB (0.2 μM, 48 h) inhibited cell proliferation of MDA-MB-231 **(H)** and MDA-MB-468 **(I)** cells. Data were presented as mean ± SD, $^{**}P < 0.01$, $^{***}P < 0.001$.

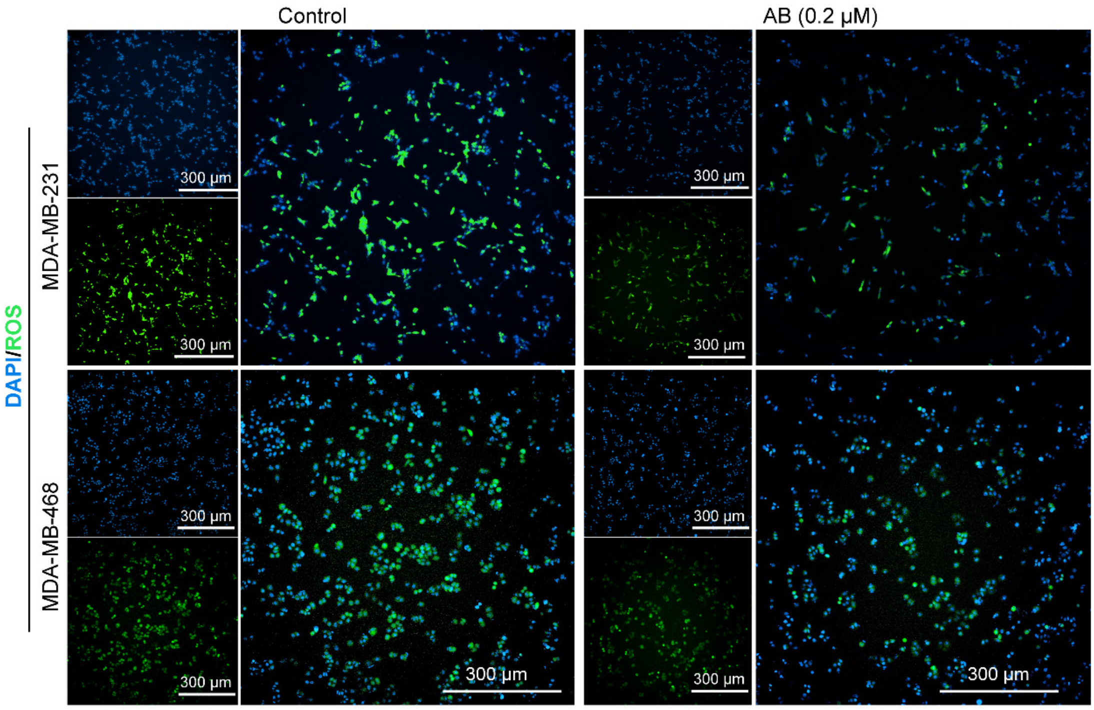

Figure S11. ROS detection after AB treatment. The immunofluorescence-based assay showed that AB (0.2 μM) inhibited ROS production in MDA-MB-231 and MDA-MB-468 cells. Data were presented as mean ± SD, $^{**}P < 0.01$, $^{***}P < 0.001$ versus the control group.

A

Protein name	Prognosis	Expression	Ratio of proteomics	DARTS (Ratio)	
				Intracellular	Extracellular
VDAC1	Disadvantage	High	2.1	2.2	1.5
HSP90AA1	No	High	1.2	1.3	2.2
PICH	Disadvantage	High	0.8	0.3	0.3
PLK1	No	High	0.6	0.4	0.3
CORO1A	Disadvantage	High	0.5	0.5	0.2
TIMELESS	No	High	0.6	0.6	0.5

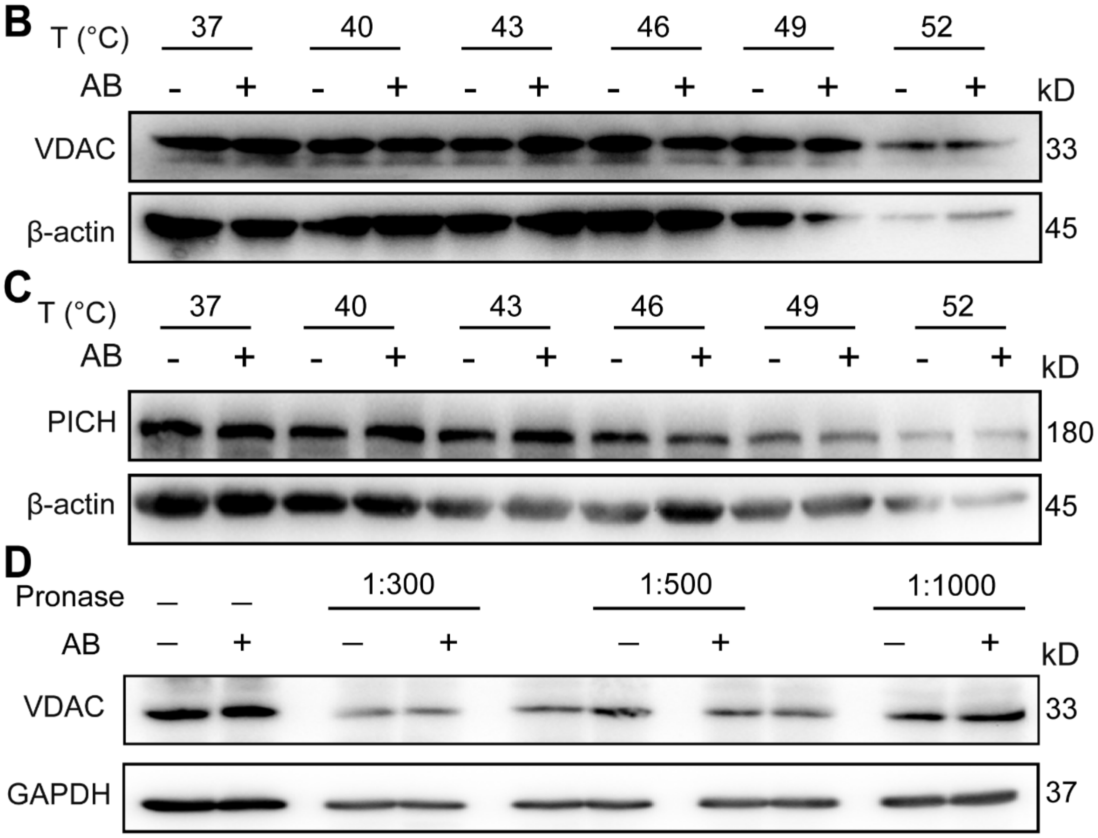

Figure S12. AB directly bound to CORO1A. **(A)** Proteins with large differences were selected both from intracellular and extracellular DARTS-MS. Extracellular CETSA assay showed that AB had little effect on protein stability of VDAC **(B)** and had no effect on protein stability of PICH **(C)**. **(D)** DARTS combined with western blot methods further showed that AB didn't induce the destabilization of VDAC during the proteolysis.

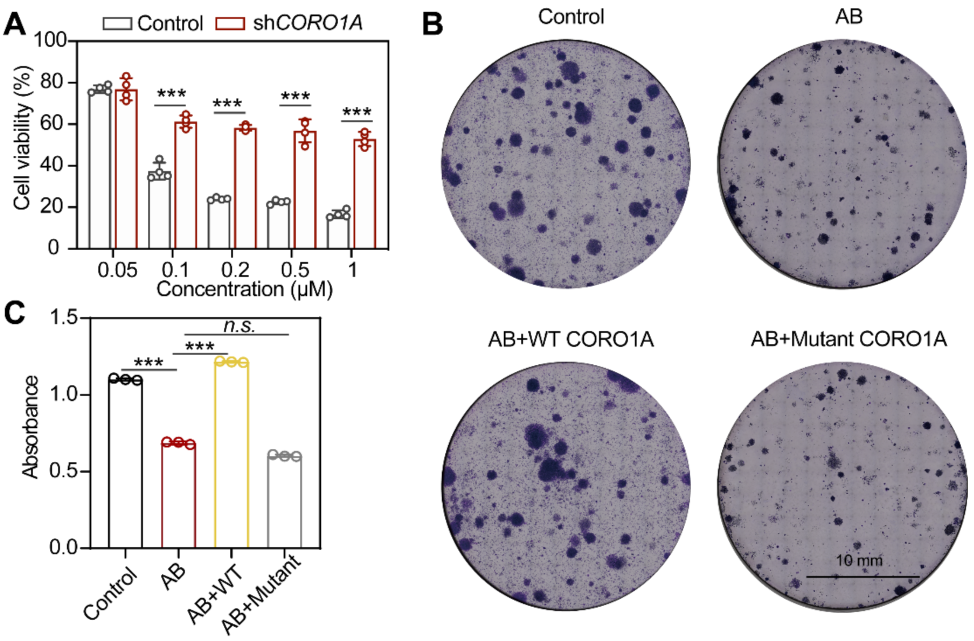

Figure S13. Cell viability and proliferation of MDA-MB-231 cells. **(A)** Cell viability of MDA-MB-231 (with or without sh*CORO1A*) cells after AB treatment for 24 h. *CORO1A* knockdown significantly alleviated the inhibition effect of AB on MDA-MB-231 cells. **(B)** Clonal formation assays showed that the inhibitory effect of AB on the proliferation of MDA-MB-231 cells can be reduced by CORO1A-WT plasmid supplementation, but the mutant CORO1A had no such effects. Data were presented as mean ± SD, ***$P < 0.001$ versus the control group. *n.s.* indicated no statistical significance.

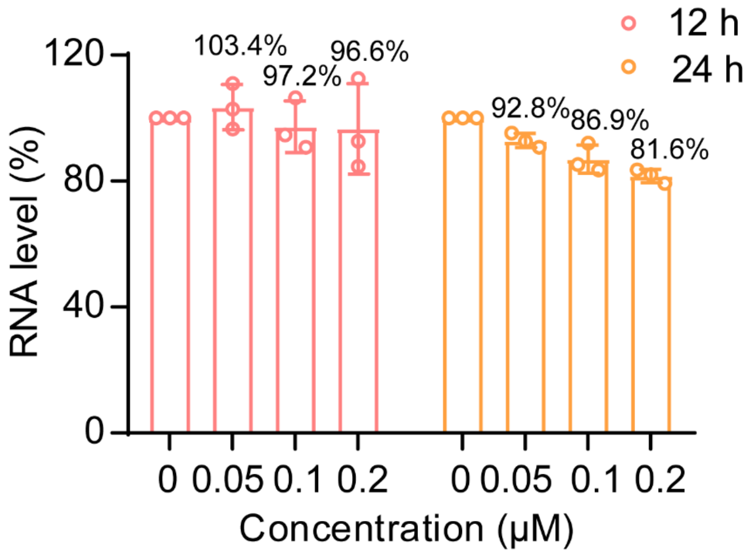

Figure S14. qPCR assays showed that AB (12 h or 24 h treatment) had no significant effect on the mRNA level of *CORO1A*.

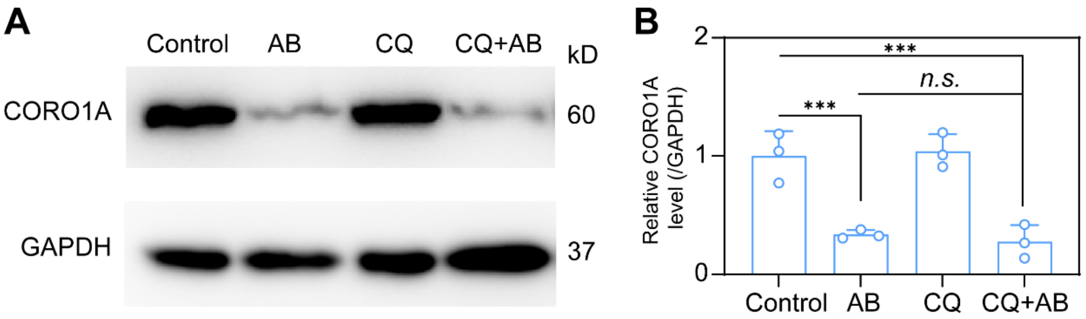

Figure S15. CORO1A detection. (A) Western blot assay showed that CQ (0.5 μM) had no significant effect on the degradation of CORO1A induced by AB (0.2 μM). The analysis was showed in **(B)**. Data were presented as mean ± SD, ****P* < 0.001 versus the control group. *n.s.* indicated no statistical significance.

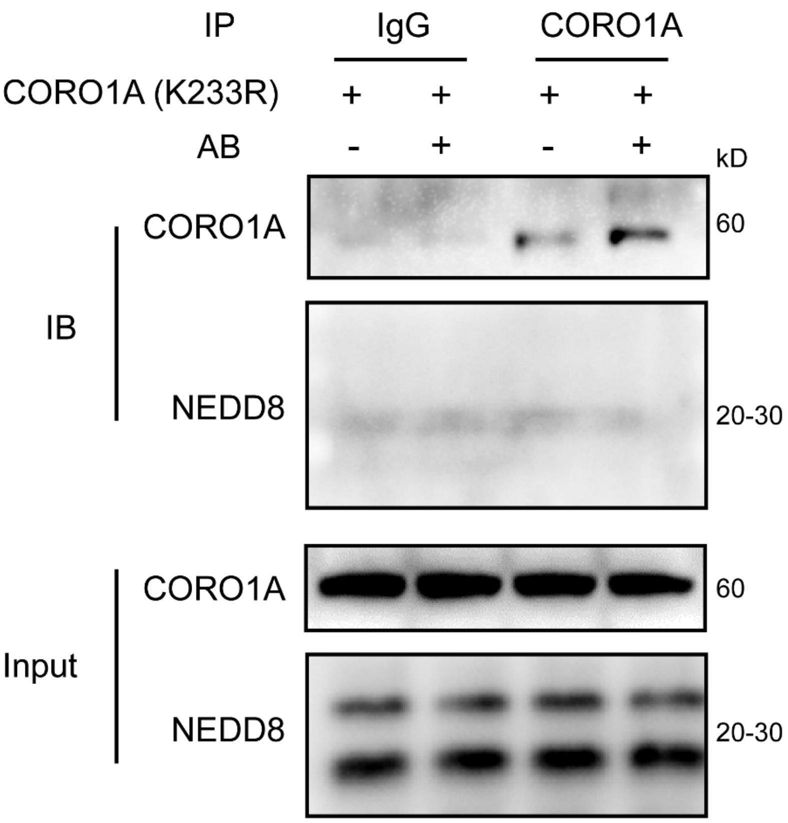

Figure S16. Co-IP assays using mutant CORO1A (CORO1A-K233R). The mutation of CORO1A (CORO1A-K233R) obviously diminished CORO1A neddylation in cells

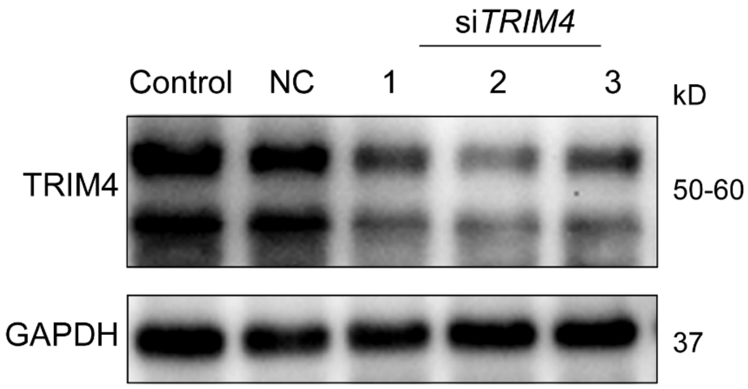

Figure S17. Protein expression of TRIM4. Western blot assay determined the knockdown of *TRIM4* in MDA-MB-231 cells using siRNA. The si*TRIM4*-2 had good effects and were used for next experiments.

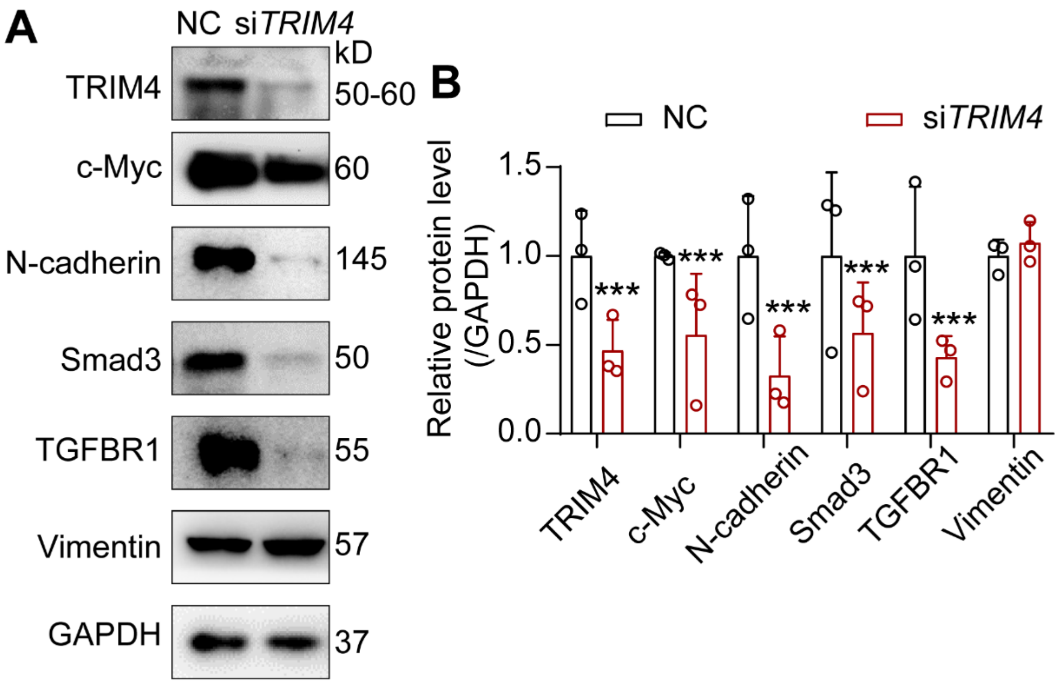

Figure S18. Knockdown of *TRIM4* decreased proteins expression in TGF-β and EMT signaling pathway. (A) Protein detection. (B) Analysis of (A). Data were presented as mean ± SD, $^{***}P < 0.001$ versus the NC group.

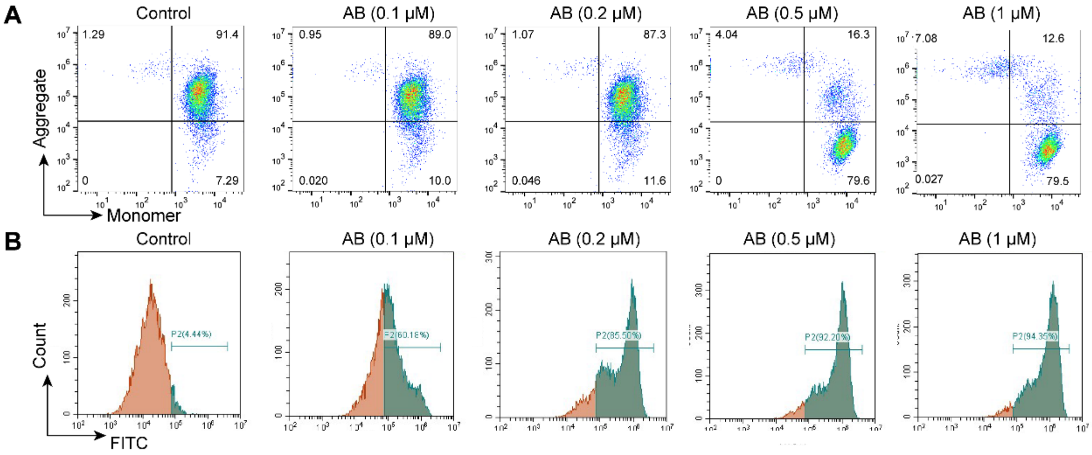

Figure S19. Detection of the mitochondrial membrane potential and calcium ion concentration after AB treatment for 24 h. **(A)** The level of mitochondrial membrane potential on MDA-MB-231 cells was significantly reduced after AB (0.2 μM) treatment for 24 h. **(B)** The calcium level on MDA-MB-231 cells was significantly increased after AB (0.2 μM) treatment for 24 h.

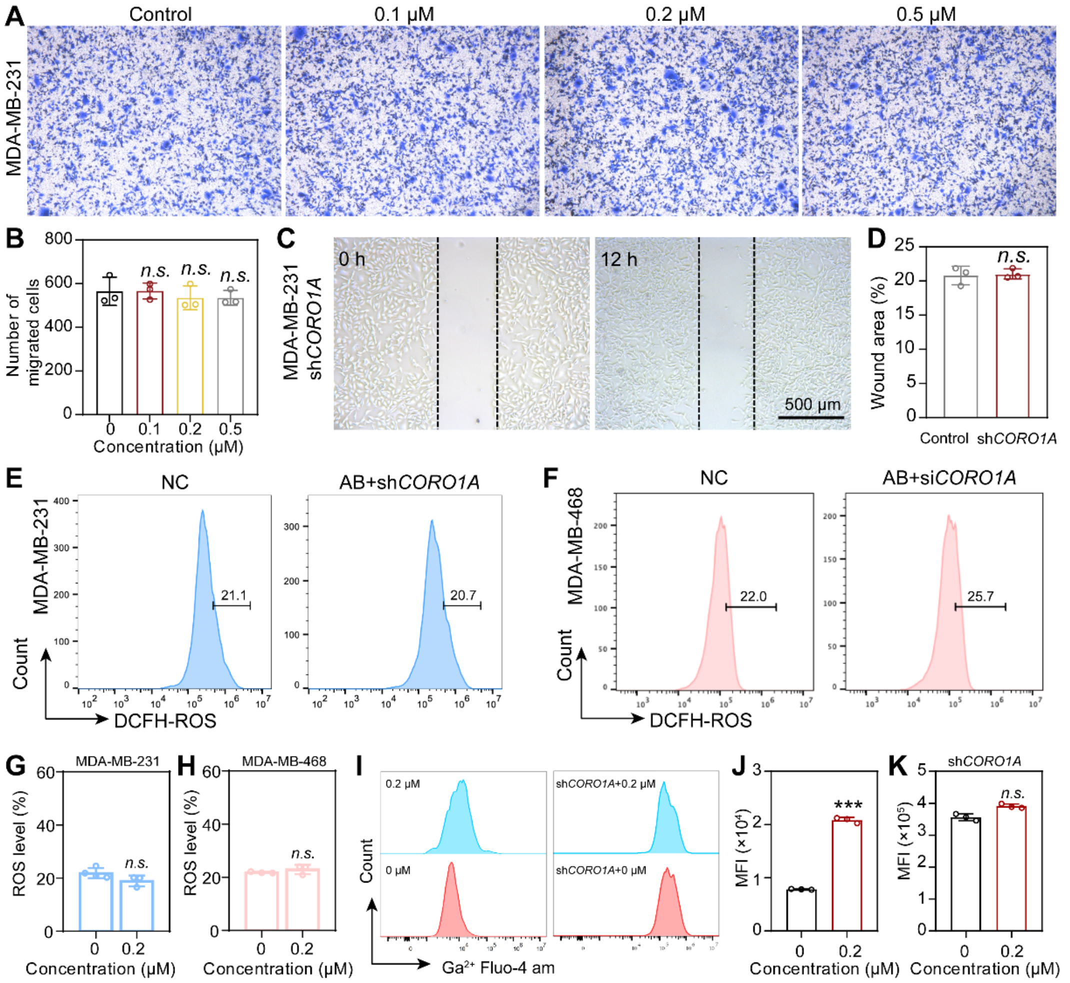

Figure S20. AB exerted anti-tumor effects through CORO1A. **(A)** *CORO1A* knockdown exhibited an alleviated migration ability of MDA-MB-231 cells after AB treatment for 24 h.

The analysis was showed in **(B)**. **(C)** *CORO1A* knockdown inhibited cell motility. The analysis was showed in **(D)**. The production of ROS induced by AB were significantly reversed on MDA-MB-231 **(E)** and MDA-MB-468 cells **(F)** after the *CORO1A* knockdown. The analysis was showed in **(G) and (H)**, respectively. **(I)** The concentration of calcium ion induced by AB was significantly reversed after *CORO1A* knockdown. The analysis was showed in **(J)** and **(K)**. Data were presented as mean ± SD, ***$P < 0.001$ versus the control group. *n.s.* indicated no statistical significance.

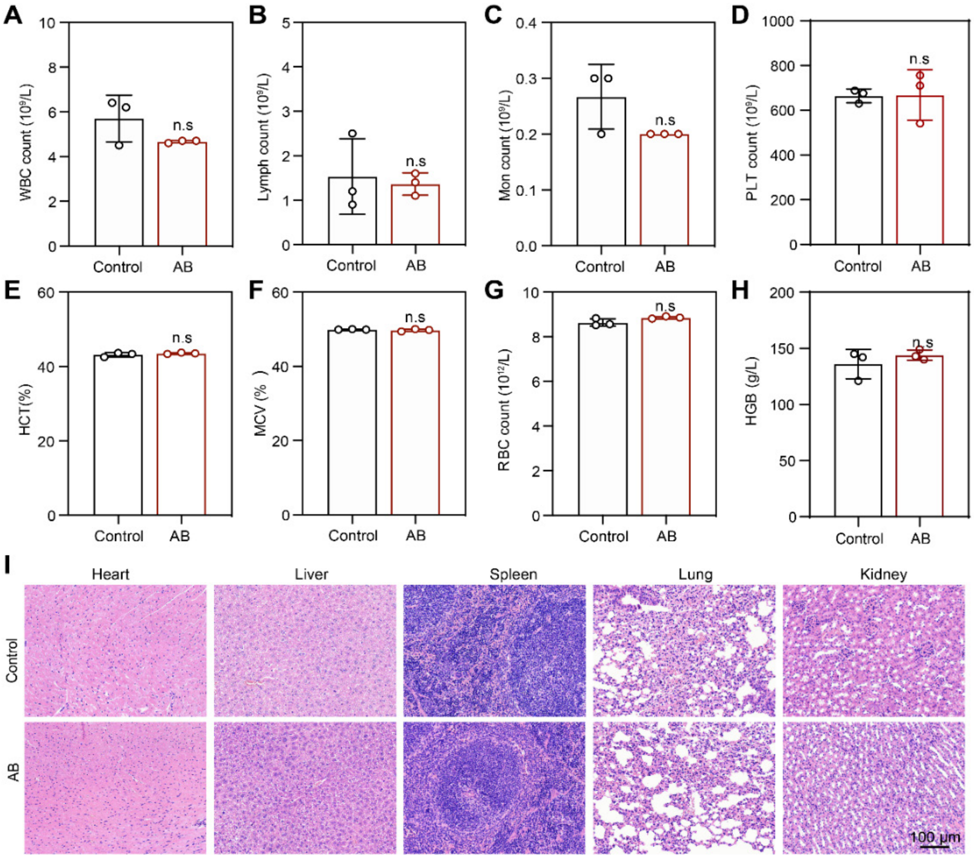

Figure S21. *In vivo* safety investigation of AB. **(A)** WBC count. **(B)** Lymph count. **(C)** Mon count. **(D)** PLT count. **(E)** HCT count. **(F)** MCV count. **(G)** RBC count. **(H)** HGB count. **(I)** Immunohistochemical staining of normal organs. Data were presented as mean ± SD, *n.s* represented no statistical significance versus the control group.

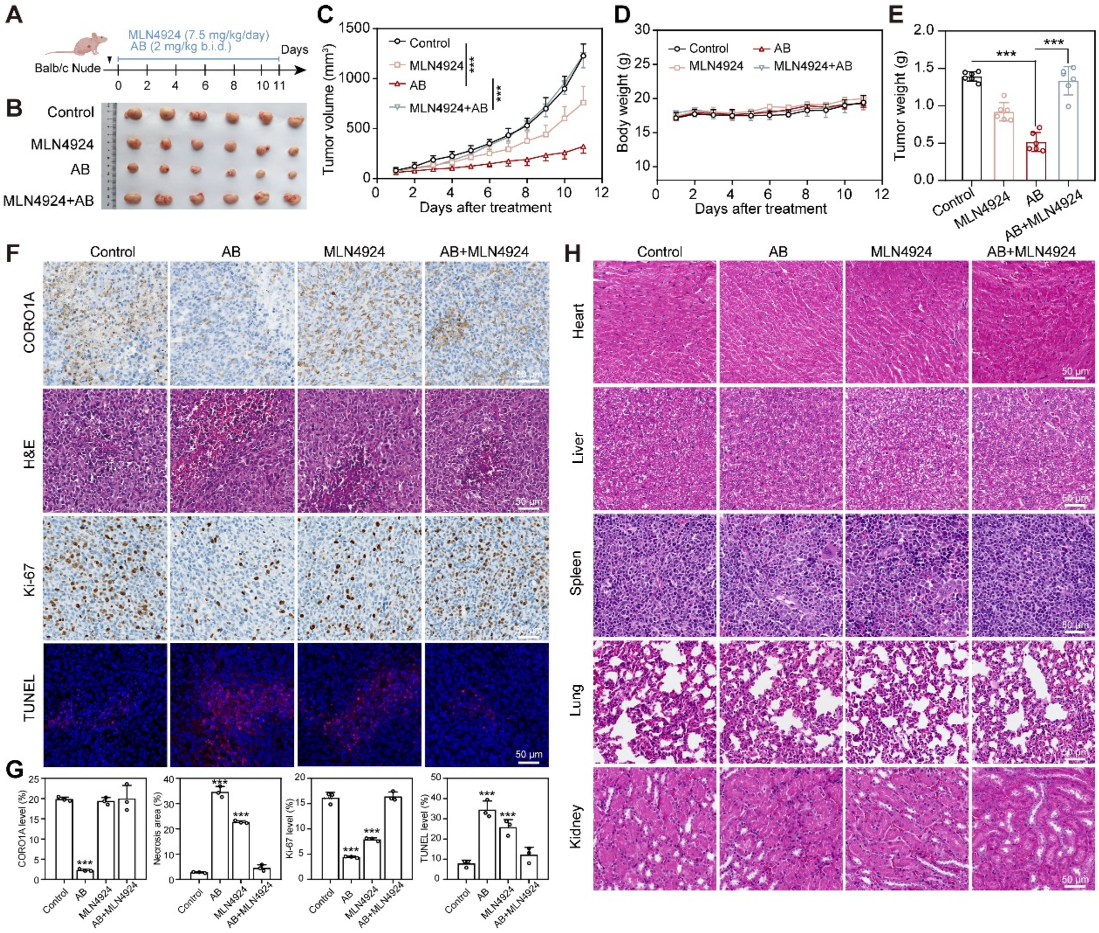

Figure S22. MLN4924 attenuated the antitumor effect of AB in TNBC tumor models. **(A)** Schematic diagram of *in vivo* study. **(B)** When mice were sacrificed, the tumors were photographed (n = 6 mice). **(C)** Tumor volume. **(D)** Body weight. **(E)** Tumor weight. **(F)** IHC staining of CORO1A expression, necrosis, proliferation, and apoptosis. **(G)** Analysis of (F). **(H)** Immunohistochemical staining of normal organs. Data were presented as mean ± SD, ***$P < 0.001$ versus the control group.

Table S1. Virtual screening for candidate compounds.

No.	Name	Score	Molecular weight (g/mol)	IC_{50} (μM)
1	Poricoic acid A	-8.76	498.70	> 100
2	Rosavin	-8.56	428.43399	> 100
3	Cichoric Acid	-8.52	474.37	6.48
4	Eupalinolide B	-8.51	462.495	> 100
5	Salvianolic acid A	-8.35	494.45	> 100
6	Brassinolide	-8.19	480.69	> 100
7	Quillaic acid	-8.16	486.69	> 100
8	Aurovertin B	-8.15	460.50	0.088
9	Salvianolic acid C	-8.15	492.44	> 100
10	Acevaltrate	-8.11	480.51	19.46
11	Kushenol I	-8.07	454.52	2.51
12	Phyllanthin	-8.07	418.53	> 100
13	Crustecdysone	-8.04	480.64	> 100
14	Schisandrin	-8.04	432.51	> 100
15	β-Elemonic acid	-8.03	454.69	> 100
